# Protein phosphatase 1 catalyzes HBV core protein dephosphorylation and is co-packaged with viral pregenomic RNA into nucleocapsids

**DOI:** 10.1371/journal.ppat.1008669

**Published:** 2020-07-23

**Authors:** Zhanying Hu, Haiqun Ban, Haiyan Zheng, Mingliang Liu, Jinhong Chang, Ju-Tao Guo

**Affiliations:** 1 Department of Experimental Therapeutics, Baruch S. Blumberg Institute, Doylestown, Pennsylvania, United States of America; 2 Biological mass spectrometry facility, Robert Wood Johnson Medical School and Rutgers, The State University of New Jersey. Piscataway, New Jersey, United States of America; 3 Institute of Medicinal Biotechnology, Chinese Academy of Medical Sciences & Peking Union Medical College, Tian-Tan Xi-Li, Beijing, China; The Pennsylvania State University College of Medicine, UNITED STATES

## Abstract

Hepatitis B virus (HBV) replicates its genomic DNA *via* viral DNA polymerase self-primed reverse transcription of a RNA pre-genome in the nucleocapsid assembled by 120 core protein (Cp) dimers. The arginine-rich carboxyl-terminal domain (CTD) of Cp plays an important role in the selective packaging of viral DNA polymerase-pregenomic (pg) RNA complex into nucleocapsid. Previous studies suggested that the CTD is initially phosphorylated at multiple sites to facilitate viral RNA packaging and subsequently dephosphorylated in association with viral DNA synthesis and secretion of DNA-containing virions. However, our recent studies suggested that Cp is hyper-phosphorylated as free dimers and its dephosphorylation is associated with pgRNA encapsidation. Herein, we provide further genetic and biochemical evidence supporting that extensive Cp dephosphorylation does take place during the assembly of pgRNA-containing nucleocapsids, but not empty capsids. Moreover, we found that cellular protein phosphatase 1 (PP1) is required for Cp dephosphorylation and pgRNA packaging. Interestingly, the PP1 catalytic subunits α and β were packaged into pgRNA-containing nucleocapsids, but not empty capsids, and treatment of HBV replicating cells with core protein allosteric modulators (CpAMs) promoted empty capsid assembly and abrogated the encapsidation of PP1 α and β. Our study thus identified PP1 as a host cellular factor that is co-packaged into HBV nucleocapsids, and plays an essential role in selective packaging of the viral DNA-polymerase-pgRNA complex through catalyzing Cp dephosphorylation.

## Author summary

Selective packaging of pregenomic RNA by core protein dimers into nucleocapsid is a key step of HBV replication and is subjected for the regulation by multiple viral and host cellular factors. HBV core protein phosphorylation and dephosphorylation play an essential role in HBV genome replication. However, the cellular kinases and phosphatases responsible for the biochemical events remain elusive. Identification of cellular protein phosphatase 1 as a host cellular factor catalyzing core protein dephosphorylation and facilitating viral pregenomic RNA packaging into nucleocapsids sheds new light on the molecular mechanism of HBV replication and development of therapeutics to cure chronic HBV infection.

## Introduction

Hepatitis B virus (HBV) chronically infects 257 million people worldwide and causes approximately 680 thousands deaths annually due to cirrhosis, hepatocellular carcinoma and liver failure [1]. Currently available antiviral therapeutics can efficiently suppress viral replication and reduce viral load, but fail to resolve the chronic HBV infection in the vast majority of treated patients [2, 3], primarily due to the failure of eliminating the covalently closed circular (ccc) viral DNA in the nuclei of infected hepatocytes and restoration of a functional immune response against the virus [4, 5]. Further understanding the molecular mechanisms underlying the persistent infection of HBV in hepatocytes and host immune tolerance to HBV infection are essential for the rational development of novel therapeutics to cure chronic hepatitis B [6].

HBV is the prototype member of *Hepadnaviridae* family and contains a 3.2 kb, partially double-stranded, relaxed circular (rc) DNA genome, which is replicated in the infected hepatocyte *via* viral DNA polymerase self-primed reverse transcription of a RNA pre-genome [7]. Briefly, HBV infects a hepatocyte by binding to its cellular receptor, sodium taurocholate cotransporting polypeptide (NTCP), and delivering the viral nucleocapsid into the cytoplasm *via* endocytosis [8]. The viral rcDNA genome in nucleocapsid is then imported into the nucleus and converted into an episomal covalently closed circular (ccc) DNA to serve as a template for transcription of viral RNAs [9]. A longer than genome-length viral RNA, designated as pregenomic (pg) RNA, serves not only as the mRNA for core protein (Cp) and DNA polymerase (Pol), but also as a template for reverse transcriptional DNA replication. Binding of Pol to the stem-loop structure (ε) at the 5’ terminus of pgRNA initiates the packaging of Pol-pgRNA complex by 120 copies of Cp dimers to form a nucleocapsid, where Pol converts the pgRNA to a single-stranded DNA and then rcDNA [10–12]. In addition, Cp dimers can also self-assemble into “empty” capsids devoid of Pol-pgRNA in infected hepatocytes [12, 13]. Interestingly, both empty capsids and nucleocapsids can be enveloped by membrane studded with three viral envelope proteins and secreted as incomplete (or genome-free) and complete virions, respectively [14].

As illustrated in S1 Fig, HBV Cp is a 183-residue polypeptide containing a N-terminal assembly domain (NTD, residues 1–140) and a C-terminal arginine-rich nucleic acid-binding domain (CTD/ARD, residues 150–183), which are linked by a hinge region of 9 amino acid residues [12]. The CTD contains seven conserved serines or threonine that can be dynamically phosphorylated and dephosphorylated during the viral replication cycle [15–20]. Previous phosphomimetic mutagenesis studies indicated that the CTD phosphorylation, particularly at S162 and S170, is required for pgRNA packaging, whereas the CTD dephosphorylation is associated with viral DNA synthesis and secretion of virions [21–24]. However, we recently developed a Western blot assay that can resolve Cp based on its phosphorylation status and found that HBV Cp is hyperphosphorylated in free dimers and empty capsids, but is hypophosphorylated in pgRNA- and DNA-containing nucleocapsids [25]. These results indicate that Cp dephosphorylation may occur during pgRNA encapsidation. Although several cellular kinases, including SRPK1 [26, 27], PKA [28], PKC [29, 30], PLK1[31] and CDK2 [32], had been shown to phosphorylate some or all the seven putative phosphoacceptor residues in the CTD in *in vitro* biochemical assays or when co-expressed with Cp in E. *coli*, their roles in Cp phosphorylation in infected hepatocytes have yet to be confirmed. The cellular protein phosphatases that catalyze the Cp dephosphorylation have not been identified.

In our efforts to further understand the molecular mechanism of HBV pgRNA encapsidation and its relationship with Cp dephosphorylation, we have now obtained additional genetic and biochemical evidence indicating that HBV Cp dephosphorylation does occur at multiple sites during pgRNA packaging and is catalyzed by cellular protein phosphatase 1 (PP1). Interestingly, the PP1 catalytic subunits α and β are also co-packaged with viral pgRNA and DNA polymerase into nucleocapsids.

## Results

### Cp dephosphorylation occurs during pgRNA-containing nucleocapsid assembly

**Fig 1:**
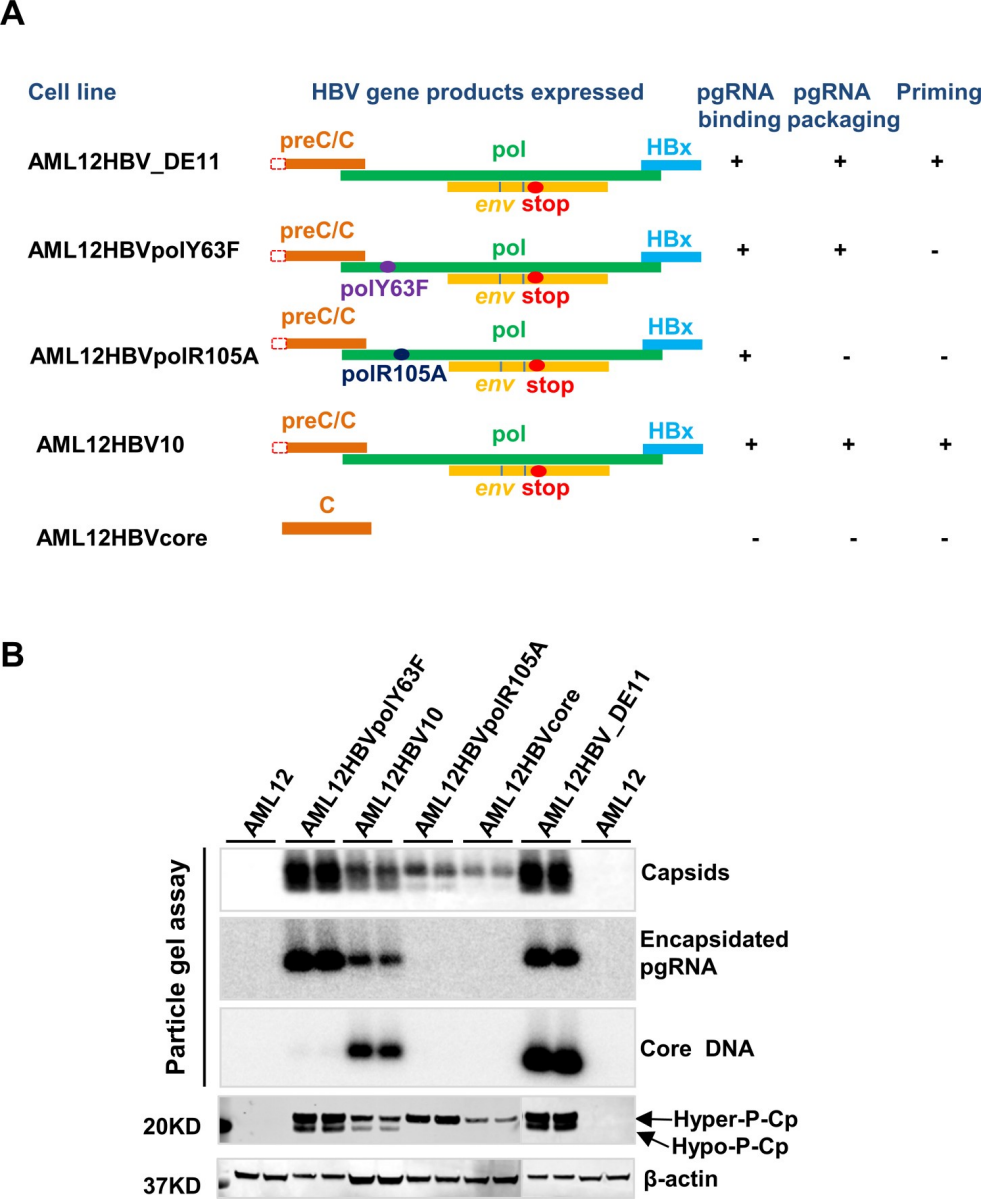
HBV Cp dephosphorylation is associated with pgRNA packaging. **(A)** AML12-derived stable cells with integrated HBV transgene that tet-off inducible expression HBV pgRNA or Cp ORF. The mutation(s) in each of the transgenes are highlighted. The biological property of the transgene-encoded polymerase, including the ability to support pgRNA-binding, pgRNA packaging and DNA synthesis, are indicated. **(B)** All the cell lines were cultured in the absence of tet for 3 days. Intracellular HBV capsids were resolved by electrophoresis in 1.8% native agarose gel and transferred on a Nylon membrane. HBV capsids were probed by a rabbit polyclonal antibody against HBV Cp (Dako). The encapsidated pgRNA and core DNA were detected by hybridization with an α-^32^P-UTP labeled full-length negative-strand and positive-strand HBV RNA probe, respectively. HBV Cp expression and phosphorylation status were determined by a Western blot assay with antibody HBc170A. β-actin served as a loading control.

Our previous studies showed that Cp is hyperphosphorylated in free dimers and empty capsids, but is hypophosphorylated in pgRNA- and DNA-containing nucleocapsids. Moreover, we found that inhibition of pgRNA packaging by heat shock protein 90 (HSP90) ATPase inhibitor (17-dimethylamino geldanamycin) and HBV core protein allosteric modulators (CpAMs) abolished hypophosphorylated Cp, and induced the assembly of empty capsids with hyperphosphorylated Cp [25]. Our results thus indicate that Cp dephosphorylation is associated with the assembly of pgRNA-containing nucleocapsids, but not empty capsids. In order to further investigate the biological function and molecular mechanism of Cp dephosphorylation in pgRNA packaging, we determined Cp phosphorylation status in a panel of five mouse hepatocyte-derived stable cell lines that tet-off inducibly express wild-type Cp alone (AML12HBVcore), pgRNA that encodes wild-type Cp as well as wild-type Pol (AML12HBV10, AML12HBVDE11) or mutant Pol that are deficient in pgRNA packaging (AML12HBVpolR105A) or priming of minus strand DNA synthesis (AML12HBVpolY63F) (Fig 1A) [33, 34]. Due to the strong tendency of Cp dimers to self-assemble into empty capsids, pgRNA- and DNA-containing nucelocapsids are usually the minor population of total capsids in HBV replicating cells [13]. Although the regular agarose gel electrophoresis-based particle gel assay cannot efficiently separate the empty capsids and nucleocapsids, the viral RNA and DNA contents of nucleocapsids can be detected by hybridization with a strand-specific probe [25, 35]. As anticipated, expression of Cp alone or Cp with R105A mutant Pol assembled only empty capsids (Fig 1B). On the contrary, expression of Cp with wild-type Pol and PolY63F mutant protein assembled not only empty capsids, but also pgRNA-containing nucleocapsids. However, only expression of pgRNA-encoding wild-type pol supported pgRNA packaging and subsequent viral DNA synthesis (AML12HBV10 and AML12HBVDE11). Interestingly, except for AML12HBVcore and AML12HBVpolR105A cell lines that only assembled empty capsids and contained only hyper-phosphorylated Cp, all the other cell lines that can assemble not only empty capsids, but also pgRNA-containing nucleocapsids had both hyper- and hypo-phosphorylated Cp. These results further demonstrated the tight association between Cp dephosphorylation and assembly of pgRNA-containing nucleocapsids, but not empty capsids and thus suggest that Cp dephosphorylation most likely takes place during pgRNA packaging.

### PP1 is required for capsid protein dephosphorylation and pgRNA encapsidation

**Fig 2:**
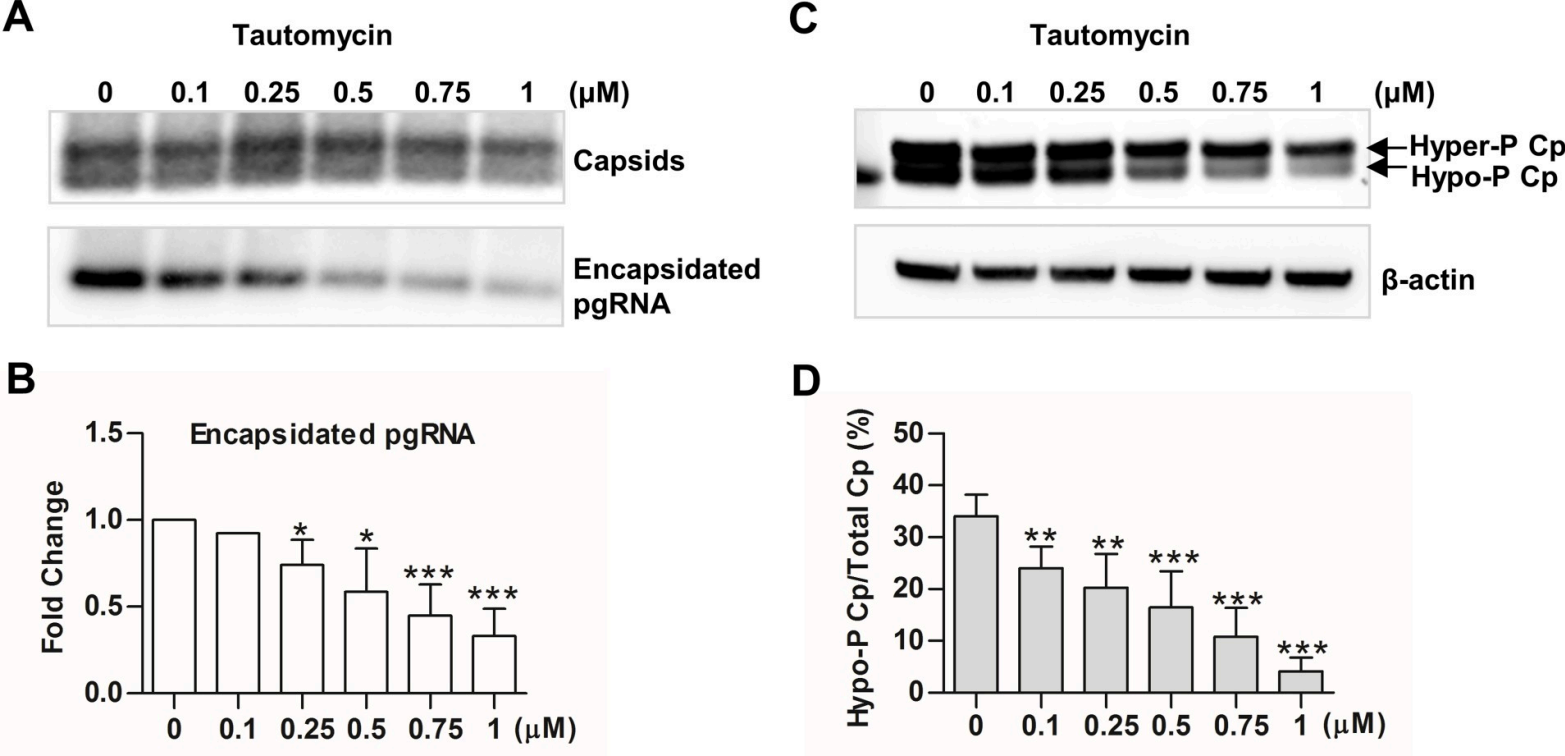
Tautomycin inhibits pgRNA encapsidation and Cp dephosphorylation. AML12polY63F cells were cultured in the absence of tet for 4 h and then mock-treated or treated with the indicated concentrations of tautomycin for 24 h. (**A**) Intracellular capsids and encapsidated pgRNA were detected by a particle gel assay. (**C**) Cp phosphorylation status was determined by a Western blot assay. (**B** and **D**) The band intensity of capsids, encapsidated RNA as well as hyper- and hypo-phosphorylated Cp in panels A and C were quantified by Gelpro32 software. The amount of encapsidated pgRNA was normalized to the amount of total capsids in each sample and presented as a fraction of the amount in mock-treated cells (B) The extent of Cp dephosphorylation was expressed as the percentage of hypophosphoryated Cp in total Cp for each sample (D). Data were derived from three independent experiments. * *P*<0.05, ** *P* <0.01, *** *P* <0.001, versus mock-treated cells (one-way ANOVA).

In order to identify host cellular phosphatase(s) that catalyze Cp dephosphorylation during pgRNA encapsidation, we first determined the effects of phosphatase inhibitors on Cp dephosphorylation in AML12HBVpolY63F cells. The results showed that tautomycin, an inhibitor of protein phosphatase 1 (PP1) and 2A (PP2A) [36], significantly reduced the percentage of hypo-phosphorylated Cp in total Cp and reduced the amounts of encapsidated pgRNA in a concentration-dependent manner (Fig 2). However, the PP2A-specific inhibitor LB-100 did not affect Cp dephosphorylation and pgRNA encapsidation (S2 Fig) [37]. We, therefore, speculated that PP1 is most likely the enzyme that catalyzes Cp dephosphorylation during pgRNA packaging.

**Fig 3:**
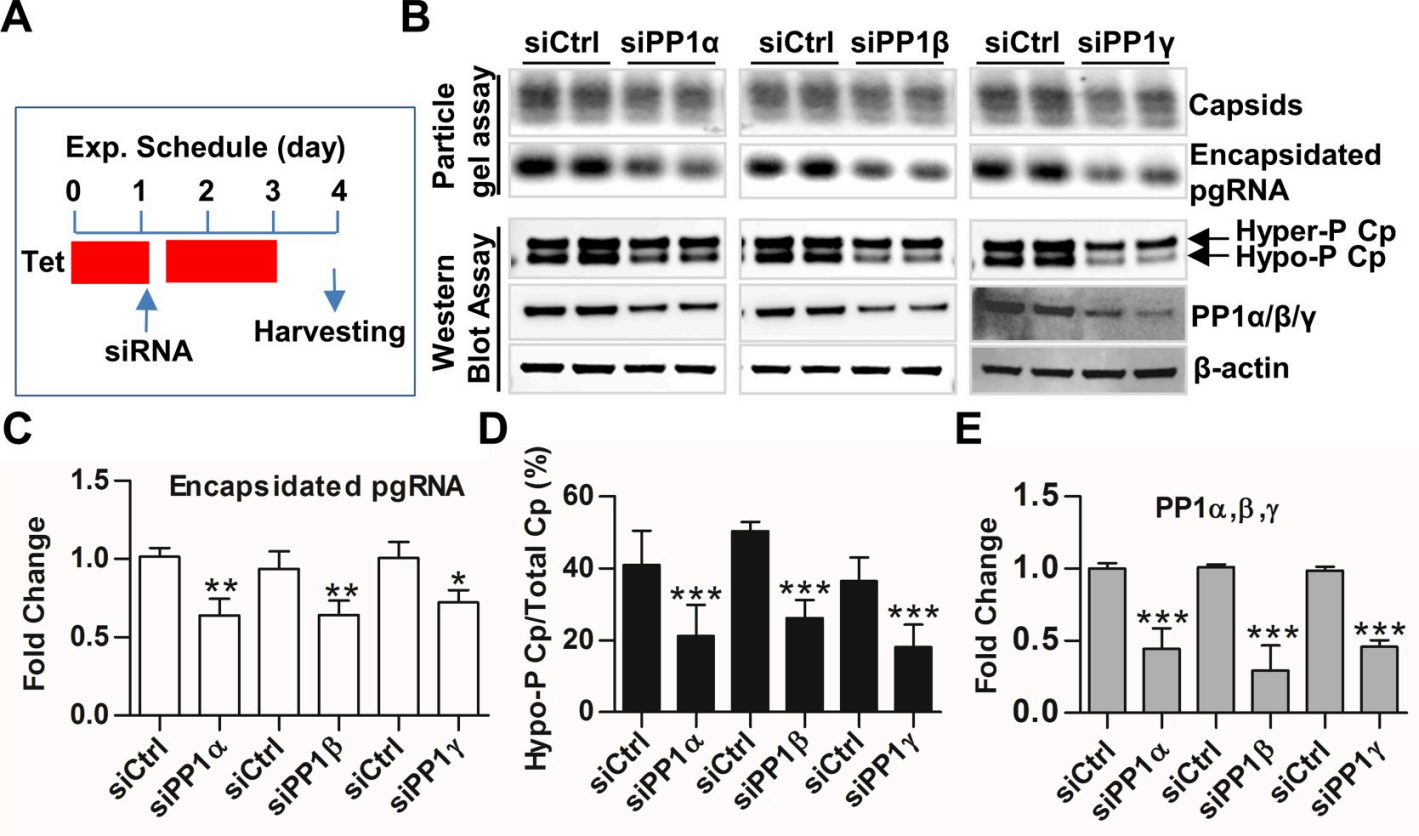
Knockdown of PP1 catalytic subunit isoforms reduced pgRNA packaging and Cp dephosphorylation. **(A)** Schematic illustration of experimental schedule. AML12HBVpolY63F cells were cultured in the presence of tet and transfected with 10 pmol siRNA targeting one of the PP1 catalytic isoforms or control siRNA by using Lipofectamine 2000 at 24 h post seeding. Six hours later, cells continued to be cultured in the presence of tet. At 48 h post transfection, cells were cultured in the absence of tet for another 24 h to allow pgRNA transcription and subsequent nucleocapsid assembly to occur. (**B**) Capsids and encapsidated pgRNA were detected by particle gel assay. The hyper/hypo-phosphorylated Cp and PP1 catalytic isoforms were detected by Western blot assays. β-actin served as a loading control. (**C** to **E**) The band intensity of capsids, encapsidated RNA, hyper- and hypo-phosphorylated Cp as well as the PP1 catalytic isoforms in panel B were quantified by Gelpro32 software. The amount of encapsidated pgRNA was normalized to the amount of total capsids in each sample and presented as a fraction of the amount in cells transfected with control siRNA (C). The extent of Cp dephosphorylation was expressed as the percentage of hypophosphorylated Cp in total Cp for each sample (D). The amount of PP1 catalytic isoform in each sample were normalized to the level of β-actin and presented as a fraction of the amount in cells transfected with control siRNA (E). Data were derived from three independent experiments. * *P*<0.05, ** *P* <0.01, *** *P* <0.001, versus siCtrl (one-way ANOVA).

Mammalian genomes contain three genes encoding distinct isoforms of PP1 catalytic subunits: PP1α, PP1β and PP1γ. As illustrated in S3 Fig, the three PP1 catalytic subunit isoforms are structurally very similar and differ mainly in their N- and C-terminal regions. Although the free PP1 isoforms exhibit similarly broad substrate specificity, under physiological condition in cells, each PP1 catalytic subunit interacts with distinct regulatory proteins (subunits) to form as many as 650 distinct PP1 holoenzyme complexes that catalyze the majority of eukaryotic protein dephosphorylation in a highly regulated and selective manner [38–40]. To determine the PP1 isoform(s) that dephosphorylate Cp and regulate pgRNA encapsidation into nucleocapsids, the expression of individual PP1 catalytic subunits was knocked down by transfection of siRNA into AML12HBV10 cells. As shown in S4 Fig, each of the siRNA only specifically knocked down the expression of their targeted, but not other PP1 isoforms. Interestingly, the results presented in Fig 3 demonstrated that down-regulating each of the three PP1 isoforms in AML12HBVpolY63F cells significantly inhibited Cp dephosphorylation and pgRNA encapsidation, as indicated by the reduced amounts of encapsidated pgRNA (Fig 3C) and decreased percentage of hypo-phosphorylated Cp over total Cp (Fig 3D). In agreement with the results obtained with pharmacological inhibition of PP2A, siRNA knockdown of PP2A catalytic subunit (PP2CA) expression did not affect Cp dephosphorylation (S5 Fig). Hence, the results indicate that each of the three PP1 catalytic subunit isoforms play a non-redundant role in Cp dephosphorylation during pgRNA packaging.

### PP1α and β co-sediment with pgRNA-containing nucleocapsids, but not empty capsids

**Fig 4:**
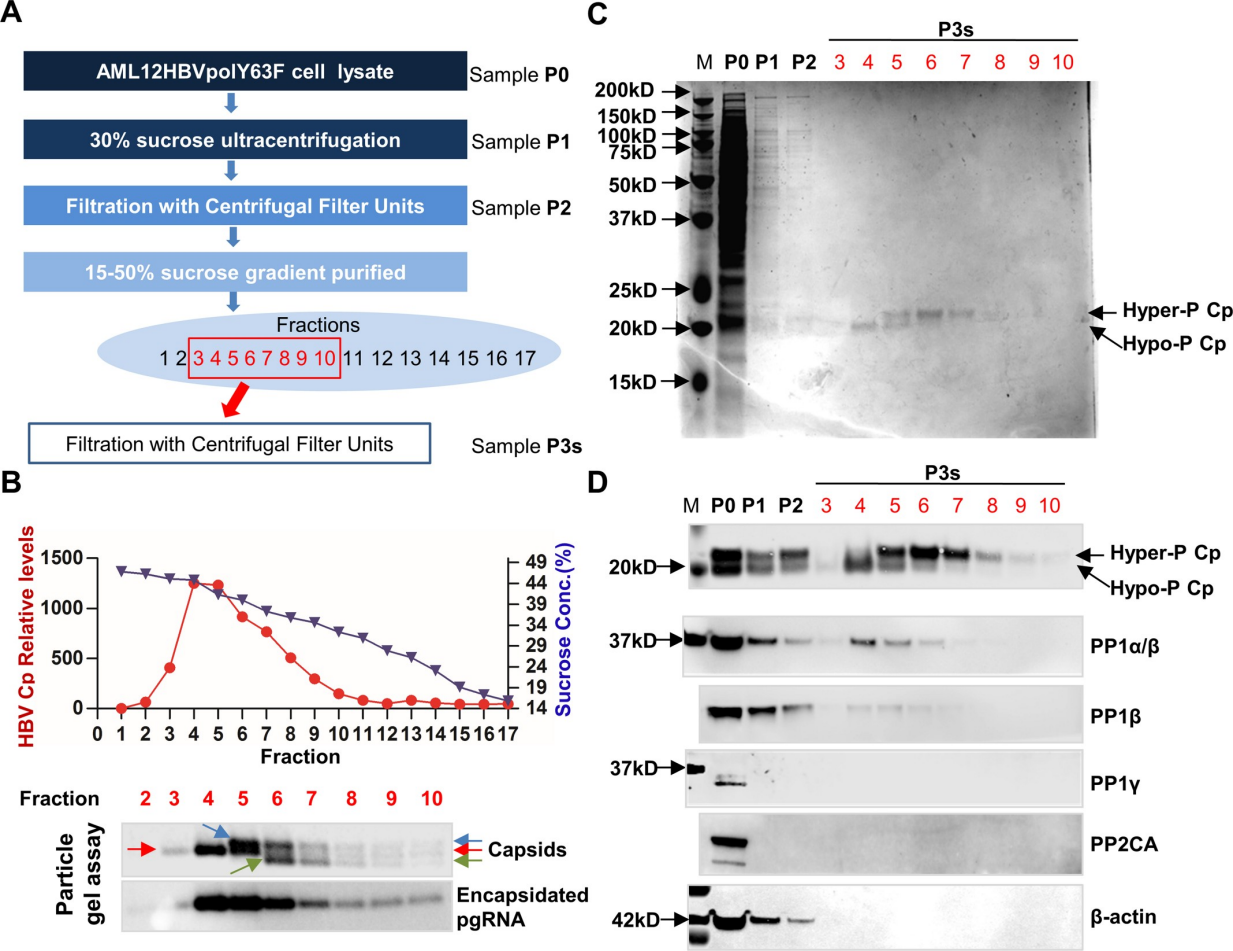
PP1α/β co-sediment with pgRNA-containing nucleocapsids and hypophosphorylated Cp. **(A)** Flowchart of HBV capsid purification. (B) Upper panel: HBV core protein/capsids in each fraction were detected by a dot immunoblot assay and quantified with Gelpro32 software. The sucrose density of each fraction was measured by Densito 30 PX. Lower Panel: The capsids in fractions 2 to10 were resolved by electrophoresis in a 1.8% agarose gel and transferred on to a membrane. Capsids were revealed by probing with an antibody against HBV core (Dako). Green and blue arrows indicate the slower and faster migrating capsids. Red arrows indicate capsids that migrate between the faster and the slower migrating capsids. Encapsidated pgRNA were determined by hybridization with an α-^32^P-UTP labeled full-length negative strand RNA as a probe. (C) Samples from each step of the capsid purification were resolved in a 12% Bis-Tris SDS-PAGE and proteins were revealed by Coomassie blue staining. (D) The desired viral and cellular proteins in the indicated fractions were detected by Western blot assays with their specific antibodies.

We next determined whether PP1 are packaged into pgRNA-containing nucleocapsids. As illustrated in Fig 4A and described in detail in Materials and Methods, we developed a four-step capsid purification procedure from the lysates of AML12HBVpolY63F cells. The sucrose gradient centrifugation analysis (Step 3) indicated that the total capsids from AML12HBVpolY63F cells peaked at fractions 4 and 5, whereas pgRNA-containing nucleocapsids sedimented slightly faster than empty capsids did and peaked at fraction 4 (Fig 4B). Coomassie blue staining indicates that the procedure can efficiently purify capsids (Fig 4C) and at least partially separate pgRNA-containing nucleocapsids and empty capsids (Fig 4B, lower panel). As extensively demonstrated in our previous report [25], while the slower and faster migrating capsids (green and blue arrows) co-sedimented with the hyperphosphorylated core proteins, the capsids (red arrow) that migrate between the faster and the slower migrating capsids appeared to co-sediment with viral pgRNA and hypophosphorylated Cp and are thus pgRNA-containing nucleocapsids (Fig 4B, lower panel, and Fig 4D, top panel). Interestingly, Western blot analysis with antibodies against PP1α and/or PP1β showed that PP1α/β co-sedimented with pgRNA-containing nucleocapsids, but not empty capsids. On the contrary, PP1γ and PP2CA were not detectable in any fractions containing empty capsids and/or pgRNA-containing nucleocapsids (Fig 4D).

### PP1α and β were packaged into nucleocapsids, but not empty capsids

**Fig 5:**
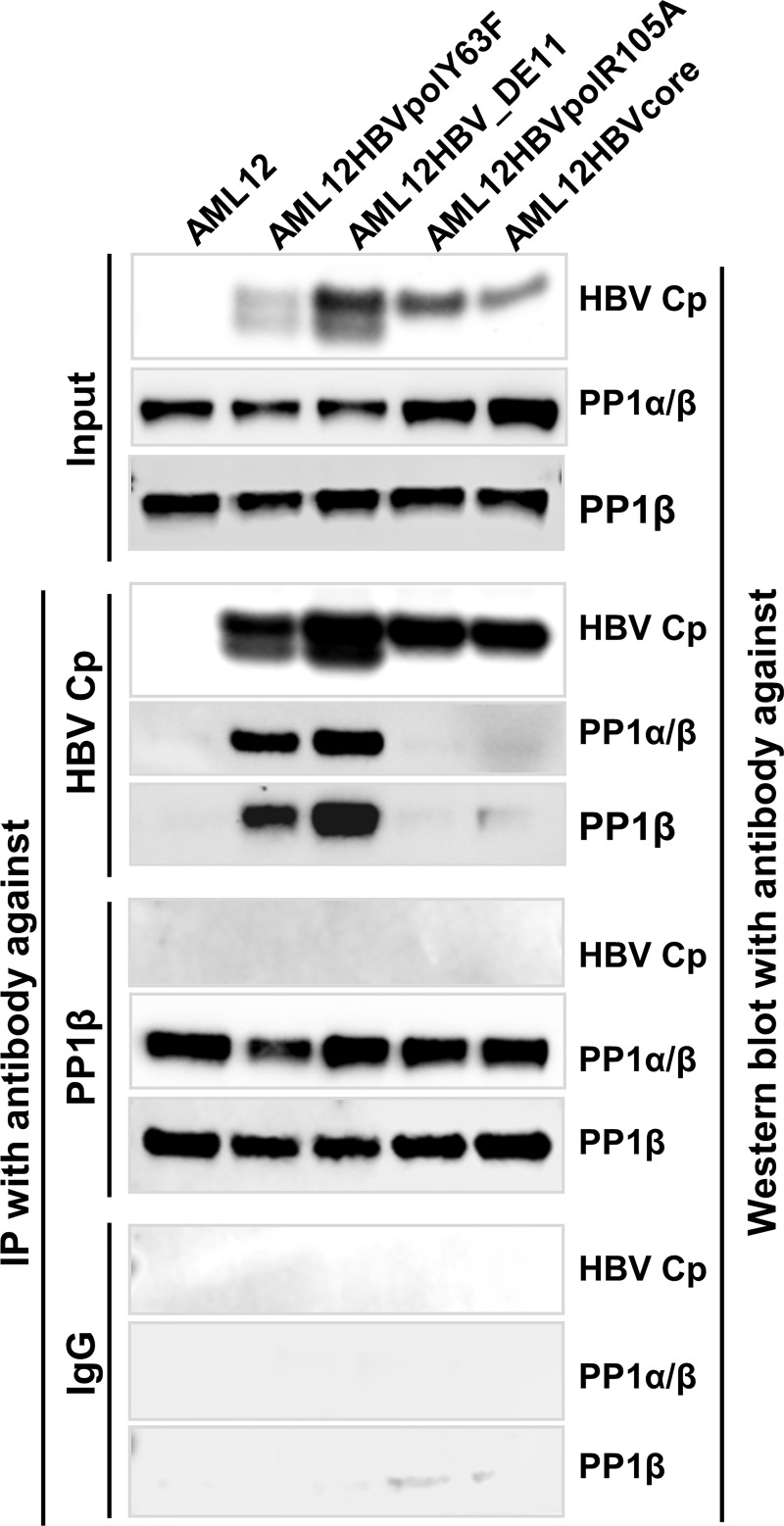
PP1α/β was encapsidated into pgRNA and DNA-containing nucleocapsids. AML12 and its derived cell lines supporting tet-off inducible accumulation of empty capsids (AML12HBVcore and AML12HBVpolR105A), pgRNA-containing (AML12HBVpolY63F) and both pgRNA- and DNA-containing (AML12HBV_DE11) nucleocapsids were cultured in the absence of tet for 2 days. The cells were lysed with IP lysis buffer. IP assays were performed with an antibody against HBV Cp, PP1β or control IgG. HBV Cp, PP1α and/or β in input cell lysates and immunocomplexes of IP were detected by Western blot assays with the corresponding antibodies.

To further investigate whether PP1α and β attach on the surface of or are packaged into the pgRNA-containing nucleocapsids, we performed immunoprecipitation assays from the lysates of AML12-derived cell lines supporting the assembly of only empty capsids, empty capsids as well as pgRNA-containing or both pgRNA- and DNA-containing nucleocapsids (Fig 1A) with antibodies against HBV capsids and PP1α/β, respectively. As shown in Fig 5, while an antibody against HBV capsids could pull down PP1α/β from the lysates of cells that produce pgRNA- or DNA-containing nucleocapsids, but not the cells that only accumulate empty capsids, an antibody against PP1β failed to pull down HBV capsids (or core protein) from the lysates of all the cell lines examined. These results demonstrated that PP1α and β do not attach on the surface of capsids, but are packaged specifically into pgRNA- and viral DNA-containing nucleocapsids.

**Fig 6:**
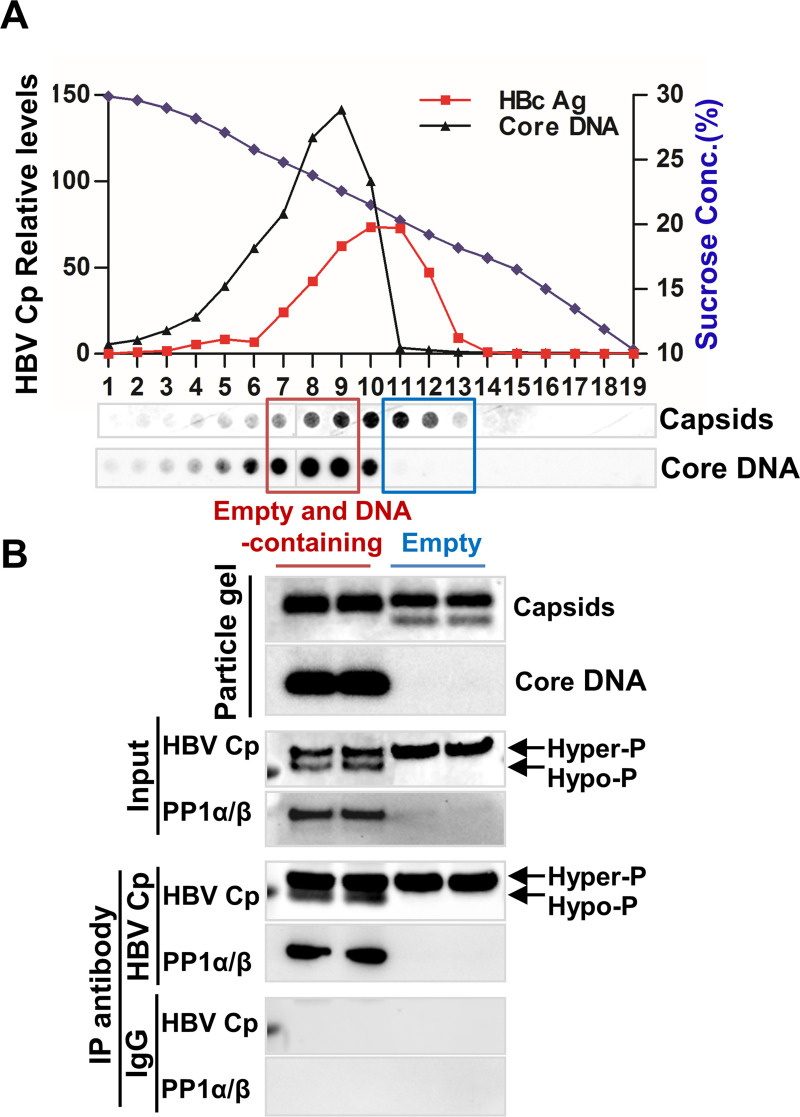
PP1α/β are encapsidated into DNA-containing nucleocapsids, but not empty capsids in HepAD38 cell. (**A**) HepAD38 cells were cultured in tet-free medium for 6 days. Intracellular capsids were separated by 15–30% linear sucrose gradient centrifugation with a Beckman SW28 rotor at 27,000 rpm for 4 h. Nineteen equal-volume fractions were collected from the top of the tube. HBV core protein/capsids in each fraction were detected by a dot immunoblot assay and core-associated HBV DNA were detected by hybridization with an α-^32^P-UTP labeled full-length positive strand RNA as a probe. Capsids and core DNA were quantified with Gelpro32 software and plotted. (**B**) Samples of fractions 7 to 9 and 11 to 13 were pooled and concentrated. The capsids and associated viral DNA were determined by particle gel assay. IP were performed with an antibody against HBV Cp or control IgG. HBV Cp, PP1α and β in input cell samples and immunocomplexes of IP were detected by Western blot assays with the corresponding antibodies.

To ascertain that PP1α and β are also packaged into nucleocapsids during HBV replication in human hepatocytes, we performed sucrose gradient centrifugation analyses of HBV capsids from HepAD38 cell lysates. Similar to that observed in mouse hepatocytes, the sucrose gradient ultracentrifugation can partially separate empty capsids (Fractions 11 to 13) and viral DNA-containing nucleocapsids (Fractions 7 to 9) (Fig 6A). While the capsids pooled from the three empty capsid fractions were primarily empty capsids, the capsids pooled from the three DNA-containing capsid fractions contained also significant amounts of empty capsids. In agreement with our previous observation [25], while the empty capsids migrated as two species with distinct electrophoresis mobility, capsids in DNA-containing capsid fractions migrate as single species (Fig 6B, top panel). Western blot analysis indicated that while hyperphosphorylated Cp existed in both fractions, hypophosphorylated Cp as well as PP1α and β were only detectable in DNA-containing capsid fractions (Fig 6B, input). Consistent with the results presented above, immunoprecipitation with an antibody against HBV core can only pull down PP1α and β in fractions with DNA-containing nucleocapsids. In agreement with these results, hypophosphorylated Cp and encapsidated PP1α and β could only be detected in HepG2 and 293T cells transfected with a plasmid containing a functional HBV replicon DNA (pCMV-HBV) supporting nucleocapsid assembly and DNA replication, but not a plasmid (pCMV-HBc) that only expresses Cp and assembles empty capsids (S6 Fig). Furthermore, immunoprecipitation assays with the lysate of 293T cells co-transfected with a HBV replicon plasmid (pCMV-HBV) and a vector plasmid or plasmid expressing a Flag/Myc tagged PP1α, PP1β or PP1ɣ showed that, similar to that observed in mouse hepatocytes, only human PP1α and β, but not PP1γ, were packaged into capsids (S7 Fig, panel A). As anticipated, similar to that observed in intracellular pgRNA/DNA-containing nucleocapsids, PP1α/β can also be detected in HBV virions and capsids derived from a HBV-positive serum (S8 Fig). Interestingly, as observed in HBV replicating hepatocytes, both hyper- and hypo-phosphorylated core proteins exist in HBV-positive serum and the hypo-phosphorylated Cp constitutes less than 10% of total Cp (S8 Fig). Because capsids in genome-free HBV virions contain hyper-phosphorylated Cp and complete HBV virions contain hypophosphorylated Cp [17], the percentage of hypo-phosphorylated Cp should reflect the percent of complete HBV virions in total HBV virion-like particles in blood. Taken together, several lines of evidence presented above strongly indicate that PP1α and β are selectively packaged into HBV nucleocapsids, but not empty capsids, in both human and mouse hepatocytes.

### CpAM treatment inhibits PP1α and β packaging into nucleocapsids

**Fig 7:**
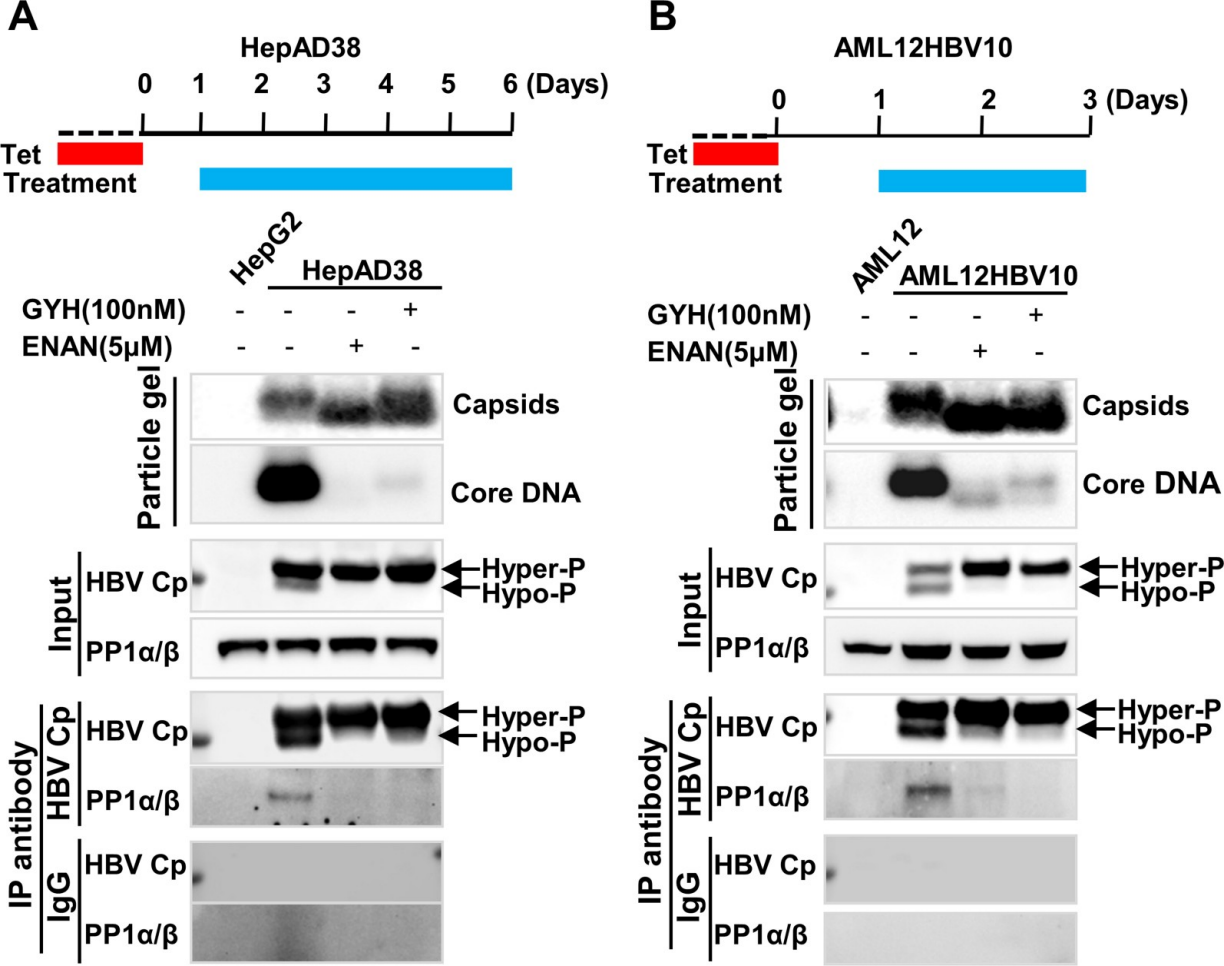
CpAMs inhibited PP1α/β encapsidated in human and mouse hepatocytes. HepAD38 cell (**A**) and AML12HBV10 cell (**B**) were cultured in the absence of tet and mock-treated or treated with the indicated concentration GYH-2-18 (GYH) and ENAN-34017 (ENAN), starting at 24 h of tet removal, for 5 and 2 days, respectively (illustrated at upper panels). Parental HepG2 and AML12 cells were used as negative controls. Intracellular capsids and HBV DNA were determined by particle gel assay. The encapsidated PP1α/β were detected by IP and Western blot assays. IP were performed with an antibody against HBV Cp or control IgG. HBV Cp, PP1α and β in input cell lysates and immunocomplexes of IP were detected by Western blot assays with the corresponding antibodies.

HBV capsid assembly is driven by the hydrophobic interaction of Cp dimer-dimer interfaces [41]. Binding of structurally distinct small molecular CpAMs to a hydrophobic “HAP” pocket formed between the inter-dimer interfaces alters the dimer-dimer interaction and results in assembly of morphologically “normal” capsids devoid of pgRNA [42]. In agreement with the notion that Cp dephosphorylation occurs during pgRNA packaging, we showed that treatment of human or mouse hepatocyte-derived cell lines supporting HBV replication with two different CpAMs, ENAN-34017 or GYH-2-18 (S9 Fig) efficiently induced the formation of capsids with slightly faster electrophoretic mobility in native agarose gel and inhibited viral DNA replication (Fig 7, particle gel assay), presumably due to inhibition of pgRNA encapsidation [35, 43]. As anticipated, CpAM treatment also abolished hypophosphorylated Cp (Fig 7, Western blot assay, Input). In agreement with its role in Cp dephosphorylation and pgRNA encapsidation, CpAM treatment prevented the encapsidation of PP1α and β, as indicated by immunoprecipitation assays with an antibody against HBV capsids (Fig 7). Similar results were also obtained with GYH treatment of AML12HBVpolY63F cells (S10 Fig).

### Cp is extensively dephosphorylated at multiple sites during pgRNA packaging

**Fig 8:**
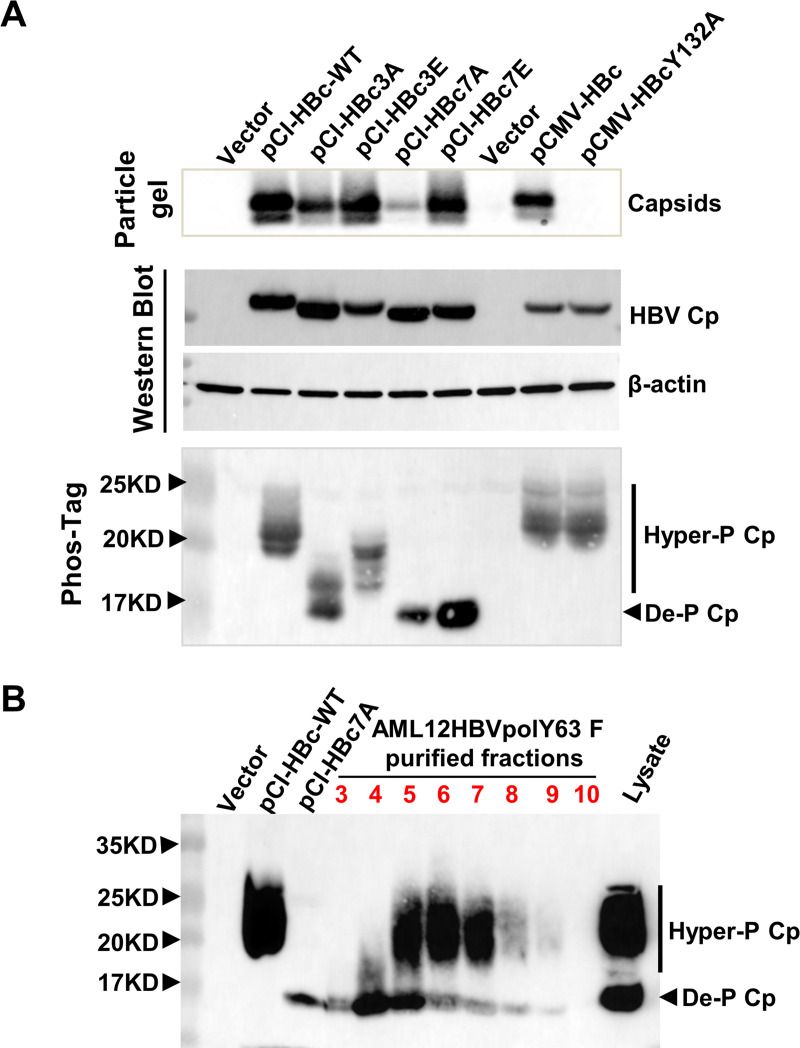
Phos-tag gel electrophoresis analysis of HBV Cp phosphorylation status. (**A**) 293T cells were transfected with the indicated plasmids and lysed with lysis buffer at 3 days post transfection. Intracellular capsids were determined by particle gel assay. HBV Cp in total cell lysates were resolved by electrophoresis in a NuPAGE 12% Bis-Tris Protein Gel and detected by a western blot assay. β-actin served as a loading control ((lower panel). The same samples were resolved by phos-tag gel electrophoresis and Cp was detected by Western blot assay with antibody HBc-170A. (**B**) The lysates of pCI-HBc and pCI-HBc-7A transfected 293T cells and total lysates of AML12HBVpolY63F cells as well as samples of sucrose gradient centrifugation fractions 3 to 10 described in Fig 4 were resolved by a 12.7% Phos-tag gel electrophoresis and proteins were transferred onto a PVDF membrane. HBV Cp was detected with antibody HBc-170A.

Although our Western blot assay can resolve Cp into hyper- and hypo-phosphorylated species, due to the existence of multiple phosphorylation sites and potential heterogeneity of phosphorylation among Cp molecules in the free dimers and different forms of capsids, more accurate analyses of the phosphorylation status of Cp should provide further molecular insights on the regulation of capsid assembly and pgRNA packaging in hepatocytes. Taking the advantage of Phos-tag gel electrophoresis technology that can resolve proteins based on the extent of their phosphorylation, we first examined Cp phosphorylation status in 293T cells transfected with plasmids expressing wild-type or distinct mutant Cp. As anticipated, except for Y132A mutant Cp that is deficient in capsid assembly [44], expression of all other Cp proteins resulted in the assembly of capsids in the cells (Fig 8A, particle gel assay). In agreement with our previous report [25], Western blot assay showed that Cp with substitution of three major (HBc-3A) or all seven (HBc-7A) phosphoacceptor sites in the CTD with alanine to mimic the partially or completely dephosphorylated Cp migrated slightly faster than wild-type or Y132A mutant Cp (Fig 8A). Phos-tag gel electrophoresis and Western blot assay detected both HBc-7A and HBc-7E as a single species with the fastest mobility, which should represent the mobility of completely dephosphorylated Cp. However, HBc-3A and HBc-3E were detected as two to three distinct species with slower mobility, suggesting the heterogeneous phosphorylation at the remaining four potential phosphorylation sites in the CTD. The distinct mobility profiles between HBc-3A and HBc-3E indicated that the A and/or E substitutions altered the phosphorylation efficiency of one or multiple remaining sites in the CTD. Interestingly, both WT and Y132A Cp were detected as three recognizable bands in a background of smear, with much slower mobility, suggesting a heterogeneous hyperphosphorylation at multiple sites. The similar hyperphosphorylation profiles between WT and Y132A Cp indicate that significant dephosphorylation may not take place (Fig 8A, lower panel) during empty capsid assembly.

In order to compare the Cp phosphorylation profile between empty capsids and pgRNA-containing nucleocapsids, the total lysate of AML12HBVpolY63F cells and sucrose gradient centrifugation fractions corresponding to Fig 4 were analyzed by Phos-tag gel electrophoresis with the lysates of 293T cells expressing WT and HBc-7A Cp as positive controls. As shown in Fig 8B, as expected, both heterogeneously hyperphosphorylated and dephosphorylated Cp were detected in total cell lysates and fractions 4 to 9, only dephosphorylated Cp was detected in fraction 3, which do not contain detectable amount of empty capsids. These results indicate that at least the vast majority of Cp molecules are extensively dephosphorylated at multiple sites of the CTD during the assembly of pgRNA-containing nucleocapsids.

## Discussion

HBV replicates its genomic DNA by packaging viral pgRNA and DNA polymerase into a nucleocapsid where the pgRNA is reverse transcribed into viral DNA [5, 7]. While the specific binding of viral DNA polymerase to the stem-loop (ɛ) structure at the 5’ terminus of pgRNA plays an important role in the selective packaging of pgRNA [45], how the Pol-pgRNA complex is packaged by Cp dimers to form a nucleocapsid remains to be understood. The observation that alteration of Cp dimer-dimer interaction by CpAM treatment as well as substitutions of single amino acid at Cp dimer-dimer interfaces specifically abrogate the assembly of nucleocapsids, but not empty capsids [35, 43], implies that packaging of Pol-pgRNA complexes require unique interactions between Cp dimers. Moreover, our finding that Cp is hyper-phosphorylated in free dimers, but dephosphorylated in pgRNA-containing nucleocapsids, together with the observation that inhibition of PP1 enzymatic activity, or reducing its expression by siRNA significantly inhibited Cp dephosphorylation and pgRNA encapsidation, strongly support the hypothesis that PP1 catalyzed Cp dephosphorylation is required for, and takes place during, nucleocapsid assembly. Moreover, the result that pgRNA-containing nucleocapsids contain predominantly dephosphorylated Cp indicates that extensive Cp dephosphorylation occurs during pgRNA encapsidation. On the contrary, the similar phosphorylation profiles of WT and Y132A mutant Cp indicate that Cp dephosphorylation does not occur during assembly of empty capsids.

The extensive Cp dephosphorylation during pgRNA packaging reported herein contradicts with the previous reports that Cp dephosphorylation was associated with viral DNA synthesis [15–18]. This discrepancy is most likely because the previous studies did not examine Cp phosphorylation status of pgRNA-containing nucleocapsids [15]. Nevertheless, the requirement of Cp dephosphorylation during the assembly of nucleocapsids, but not empty capsids, further indicates that Cp dephosphorylation play an important role in selective packaging of Pol-pgRNA complex. Early mutagenesis studies indicated that despite not being required for assembly of empty capsids, the CTD of hepadnaviral Cp is required for pgRNA encapsidation [46]. Studies indicated that the arginine-rich CTD non-specifically binds nucleic acids and is responsible for encapsidation of viral and cellular RNA in both mammalian cells and *E*. *coli*. However, phosphorylation of the CTD at multiple sites efficiently reduced the nucleic acid binding activity of Cp and its ability to non-specifically encapsidate RNA [14, 47, 48]. Moreover, a recent study showed that the phosphorylation status of Cp determines the orientation of the CTD in empty capsids when expressed in *E*. *coli* [26]. These findings suggest that phosphorylation status of CTD regulates Cp interaction with pgRNA during nucleocapsid assembly. It is thus possible that while the hyperphosphorylation of Cp in free dimers prevents the non-specific interaction with cellular RNA, the sequential dephosphorylation of the CTD gradually increases Cp interaction with pgRNA during nucleocapsid formation to ensure selective packaging of Pol-pgRNA complex. In addition, dephosphorylation of Cp may also allow the CTD to organize the structure of pgRNA and facilitate the reverse transcriptional viral DNA synthesis and nucleocapsid maturation.

It is rather interesting that whereas all the three PP1 catalytic subunit isoforms appear to play an essential role in Cp dephosphorylation and pgRNA encapsidation (Fig 3), only PP1α and β are packaged into nucleocapsids. While it is possible that PP1γ catalyzes the dephosphorylation of specific Cp residue(s) in the early stage of nucleocapsid assembly and thus not packaged into nucleocapsids, the possibility that PP1γ plays an indirect role in pgRNA packaging by dephosphorylating an unidentified host factor essential for nucleocapsid assembly cannot be ruled out. Similarly, despite co-packaged into nucleocapsids, PP1α or PP1β may also exert at least some of their functions in pgRNA packaging *via* indirect mechanisms. Nevertheless, the exclusion of PP1γ from nucleocapsids may be due to its lack of interaction with a viral or cellular partner. However, our preliminary studies by immunoprecipitation assays did not reveal interaction between any PP1 isoform and Cp or Pol. Because amino acid sequence alignment of human and mouse PP1 catalytic subunit isoforms indicates that human and mouse PP1γ have a deletion of 8 or 9 amino acid residues in the C-terminal domain as compared to PP1α and β (S3 Fig), we speculated that the variable C- and/or N-terminal region of PP1 may contain structure motif(s) determining their selective encapsidation. Interestingly, deletion of either entire C-terminal variable region alone or together with deletion of a part or complete N-terminal variable region of PP1β did not compromise its packaging into nucleocapsids (S7 Fig, panel B). In addition, shuffling the C-terminal region between PP1β and PP1γ also showed that the C-terminal region of PP1γ was not responsible for the exclusion of PP1γ from nucleocapsids (S7 Fig, Panel C). These results imply that the selective encapsidation of PP1α and β, but not PP1γ, is not due to their amino acid sequence difference in N- and C-terminal regions.

Finally, although previous studies indicated that several cellular kinases are encapsidated in capsids [32], whether they are in empty capsids and/or pgRNA/DNA-containing capsids remain to be determined. If both protein kinase(s) and protein phosphatases are packaged in nucleocapsids, it will be extremely interesting to further investigate if dynamic Cp phosphorylation and dephosphorylation occurs during DNA synthesis, virion particle assembly, or disassembly of nucleocapsids upon infection of new target cells. As a preliminary effort to investigate the role of Cp dephosphorylation in viral DNA synthesis, the effects of phosphatase inhibitors on Cp phosphorylation status and viral DNA synthesis were determined in an endogenous DNA polymerase reaction *in vitro* with purified cytoplasmic HBV nucleocapsids from AML12HBV10 cells. The results indicate that either tautomycin or LB-100 did not alter Cp phsophorylation status and inhibit viral DNA synthesis (S11 Fig). These results are consistent with the results presented in Fig 8 showing that pgRNA encapsidation is associated with almost complete dephosphorylation of Cp and no further significant Cp dephosphorylation is required for viral DNA synthesis. However, because kinases, such as CDK2, are also packaged into capsids, it is possible that rephosphorylation and dephosphorylation may occur on selected residues of Cp that are escaped from our detection. Indeed, a recent report suggested that rephosphorylation of Cp at both NTD and CTD by the packaged CDK2, following CTD dephosphorylation during nucleocapsid maturation, facilitates uncoating and cccDNA synthesis by destabilizing mature nucleocapsids [49]. Obviously, the role and molecular mechanism of dynamic, site-specific Cp phosphorylation and dephosphorylation in pgRNA packaging viral DNA synthesis and uncoating remain to be further investigated.

## Materials and methods

### Cell culture

Human hepatoblastoma cell line HepG2 (HB-9065) and human embryonic kidney epithelial cell line 293T (CRL-3216) were purchased from ATCC. The HepAD38 cell line supporting tet-off inducible HBV replication [50] and immortalized mouse hepatocyte cell line AML12 [51, 52] were obtained from Christoph Seeger at Fox Chase Cancer Center, Philadelphia, PA. AML12-derived lines AML12HBV10 and AML12HBVpolY63F were established in our laboratory as reported previously [25, 53]. All the human or mouse hepatocyte-derived cells lines were cultured in DMEM-F12 medium (Invitrogen) supplemented with 10% fetal bovine serum, 100 U/ml penicillin, and 100 μg/ml streptomycin. If required, 1 μg/ml tetracycline was included in culture media to suppress HBV transgene expression. 293T cell was cultured in DMEM medium (Invitrogen) supplemented with 10% fetal bovine serum, 100 U/ml penicillin, and 100 μg/ml streptomycin.

### Chemicals

Protein phosphatase inhibitors Tautomycin and LB-100 were purchased from EMD Millipore (Cat. No. 580551) and MedKoo (Cat. No. 206834), respectively. ENAN-34017 was synthesized in house as reported previously [54]. GYH-2-18 was synthesized at Institute of Medicinal Biotechnology, Chinese Academy of Medical Sciences & Peking Union Medical College. All the compounds were dissolved in DMSO and stored at -20°C.

### Antibodies

A rabbit polyclonal antibody against HBV core protein for particle gel assay was purchased from Dako (B0586). A mouse monoclonal antibody against HBV core protein for immunoprecipitation assay was obtained from Santa Cruz (sc-52406). A rabbit polyclonal antibody (HBc-170A) against the C-terminal 14 amino acids (aa170 to 183) of HBV core protein for detecting HBV core protein phosphorylation status by Western blot assay was generated at GenScript, Piscataway, NJ, USA [25]. Information for other antibodies as following: Mouse monoclonal antibody against β-actin (CST,4967); PP1α rabbit polyclonal antibody (Sigma, PLA0154-100UL); PP1γ rabbit polyclonal antibody (Sigma, PLA0155-100UL); PP1β rabbit polyclonal antibody for Western blot assay (Proteintech, 55136-1-AP); PP1β rabbit polyclonal antibody for immunoprecipitation assay (Abcam, ab53315); PP1α+PP1β rabbit antibody HRP (Abcam, ab211372); PP2CA rabbit polyclonal antibody (Proteintech, 13482-1-AP); Mouse monoclonal anti-Myc antibody (CST,2278); Mouse anti-Flag antibody (Sigma, F1804). Mouse IgG (Ms Gamma Globulin control) (invitrogen, 31878) and Rabbit IgG (Rb Gamma Globulin control)(invitrogen, 31887) were used in IP assay as controls.

### Plasmids

pCMV-HBV expressing HBV pgRNA under the control of CMV immediate early promoter was reported previously [55–57]. The plasmids pCI-HBc-WT, pCI-HBc-3A, pCI-HBc-3E, pCI-HBc-7A or pCI-HBc-7E were gifts of Dr. Jianming Hu at Pennsylvania State University [58]. pTRE_HBV_DES and pTRE_HBV_DEpolY63F were reported previously [25, 59]. pTRE_HBV_DEpolR105A was constructed by introducing R105A substitution in polymerase into pTRE_HBV_DES by an overlapping PCR strategy. Specifically, DNA fragments were amplified first with primers F1, R105 and primers F105 and R1. A ligation PCR was performed using these two DNA fragments (1:1) as templates with F1 and R1 primers. The resulted DNA fragment was digested with *Kpn I* and *EcoR* I to replace the corresponding fragment of pTRE_HBV_DES and yielded a plasmid pTRE_HBV_DEpolR105A. In order to construct a plasmid expressing HBV Cp in a tetracycline (tet) inducible manner, HBV Cp open reading frame was amplified with primers Core F and Core R. The PCR product was restricted with *BamH I* and *HindⅢ* and cloned into *BamH I* and *HindⅢ* restricted pTRE2 to yield pTRE-HBc. Sequences of the primers are provided in S1 Table.

Human PP1α-pCMV6-entry (RC212268), PP1β-pCMV6-entry (RC201142) and pp1γ-pCMV6-entry (RC204158) plasmids with Myc and Flag tags were purchased from OriGene. Plasmids expressing PP1β with N- and/or C-terminal deletions were constructed by replacing of the authentic region with synthetic DNA fragments at Quintara Biosciences (S2 Table) through standard subcloning technology. Briefly, at first, DC fragment were restricted with *BSTE II* and *Not I* to replaced the corresponding region in *BSTE II* and *Not I* restricted PP1β-pCMV6-entry to yield plasmid pPP1β-DC with deletion of amino acids 298 to 326. Similarly, DNA fragments DN1, DN2 or DN3 were restricted with *Kpn I* and *Nhe I* to replace the corresponding region in plasmid pPP1β-DC and yield plasmids pPP1β-DN1/DC, pPP1β-DN2/DC or pPP1β-DN3/DC with deletion of amino acids 298 to 326 at C-terminus and deletion of amino acids 3–17, 3–39 or 17–39 at N-terminus (illustrated in S7 Fig). Similarly, PP1β/PP1γ chimeric and insertion of residues GM into the corresponding region of PP1β were constructed by replacing of the authentic region with synthetic DNA fragments at Quintara Biosciences (S2 Table) through standard subcloning technology. Briefly, the synthesized PP1β-γ and PP1β-GM fragments were restricted with *BSTE II* and *Not I* to replace the corresponding region in *BSTE II* and *Not I* restricted PP1β-pCMV6-entry to yield plasmids PP1β-γ and PP1β-GM. DNA fragment PP1γ-β was restricted with *Xcm I* and *Not I* to replaced the corresponding region in *Xcm I* and *Not I* restricted PP1β-pCMV6-entry to yield plasmid PP1γ-β.

### siRNA

Oligo Duplex specifically targeting mouse PP1β (Locus ID 19046), PP1α (Locus ID 19045), PP1γ (Locus ID 19047), Ppp2CA (Locus ID 19052) and universal scrambled negative control siRNA (Sictrl) (Locus ID: SR30004) were purchased from OriGene.

### Establishment of stable cell lines

AML12-derived stable cell line AML12HBV_DE11, AML12HBVpolR105A and AML12HBVcore were established by co-transfection of AML12 cells with plasmid pTRE_HBV_DES, pTRE_HBV_DEpolR105A or pTRE_HBVcore with pTet-off (Clontech) at a molar ratio of 10:1. The transfected cells were selected with 400 μg/ml G418 in the presence of 1μg/ml tetracycline. G418-resistant colonies were picked and expanded into cell lines.

### Purification of HBV capsids

Cells in a 10 cm plate were lysed with 2 mL of cell lysis buffer [10mM Tris-HCl (pH 8.0), 1mM EDTA, 0.5% NP40] at 4°C for 10 min. The cell lysates were clarified by centrifugation at 10,000 × g for 10 min at 4°C. The supernatant was loaded onto a 30% sucrose cushion and centrifuged at 46,000 rpm for 3.5 h (Beckman, Rotor SW55). Pellet was dissolved in 2 mL of TNE buffer (10 mM Tris-HCl, pH 7.4; 150 mM NaCl, 1 mM EDTA) for overnight at 4°C and then centrifuged at 10,000 × g for 10 min at 4°C. The supernatant was filtered with 100 KD cutoff Centrifugal Filter Units (Millipore, UFC810096) by centrifugation at 4000 rpm for 15 min at 4°C. The remaining solution was reconstituted with TNE buffer to 1 ml and loaded onto a 15% to 50% linear sucrose gradient in TNE buffer and spin at 27,000 rpm for 16 h (Beckman, Rotor SW28). 2 mL fractions were collected from the bottom of the centrifugation tube. 50 μL of each fraction were applied to Nylon membrane with Dot Blot Manifold for detection of HBV capsids with an antibody against HBV Cp (DAKO, B0586) and core DNA or encapsidated pgRNA by hybridization. Fractions 3–10 were concentrated and dialyzed to remove sucrose by using 100 KD Centrifugal Filter Units (Millipore, UFC810096). Briefly, The fractions were centrifuged at 4000 rpm for 15 min at 4°C and washed with 4 ml TNE buffer for 3 times. Finally, each fraction was reconstituted with 300 μL TNE buffer. HBV capsids, and encapsidated pgRNA were determined by particle gel assay (see below). HBV Cp phosphorylation status was measured by a Western blot assay described previously [25].

### Analyses of HBV Capsid assembly, pgRNA encapsidation and Cp dephosphorylation

HBV capsid assembly, pgRNA encapsidation and DNA replication were assessed by 1.8% agarose gel electrophoresis-based particle gel assay as reported previously [25, 60]. Specifically, the capsids in cell lysates were resolved in a native agarose gel (1.8%) electrophoresis and transferred onto a Nylon membrane. The capsids were detected by probing the membrane with anti-HBV Cp antibody (DAKO, B0586). Capsid-associated DNA and encapsidated RNA were detected with an α-^32^P- UTP labeled full-length HBV positive strand and negative strand RNA as a probe, respectively. Total HBV Cp was detected by regular Tris-glycine SDS-PAGE and Western blot assay with antibody HBc-170A [25]. The hyper- and hypo-phosphorylated Cp were resolved by electrophoresis in a NuPAGE 12% Bis-Tris Protein Gel (Invitrogen) and transferred onto PVDF membrane (Invitrogen). The membrane was probed with antibody HBc-170A. The bound antibody was revealed by IRDye secondary antibodies and visualized by Li-COR Odyssey system.

### Immunoprecipitation (IP) assay

Cells were lysed with IP lysis buffer (Thermo Fisher, 87788) and the lysates were cleared by centrifugation at 12,000 rpm for 10 min at 4°C. The cleared lysates were mixed with 20 μl Dynabeads Protein G beads (Invitrogen, 10004D) and incubated at 4°C for 30 min. The beads were removed and desired antibody was then added into the lysates and incubated overnight at 4 ºC. Dynabeads Protein G beads were added and incubate at 4°C for 4 h. The beads were washed with IP lysis buffer for 8 times. The immunocomplexes on beads were eluted by addition of 1x loading buffer and resolved by SDS-PAGE. The proteins were detected by Western blot assays with specified antibodies.

### Detection of PP1α/β in serum-derived HBV virion particles

HBV virions were prepared from a HBV carrier’s serum (from Bioreclamation IVT, The Complete Resources for All Biologicals) by sucrose gradient centrifugation. Two samples of healthy human serum were purchased from Sigma **(Cat. No.** H4522 and H1-100mL) and used as negative controls. Briefly, 2 ml of human serum was over-layered onto a 3 ml 30% (wt/vol) sucrose cushion in 0.15 M NaCl, 0.02 M Tris-HCl, pH 7.4, and centrifuged for 6 h at 46,000 rpm in SW55 rotor at 4 ^o^C. The supernatant fluid was removed and pellet was dissolved in 1x LDS sample buffer (Thermo Fisher, NP0007) for detection of HBV core protein and PP1α/β by Western blot assay as described above. Alternatively, 2 mL of human serum from HBV carrier or healthy individuals described above was precleared by incubation with 50 μl of Dynabeads Protein G beads at 4°C for 4 h, and then loaded onto a 3 ml of 30% (wt/vol) sucrose cushion in 0.15 M NaCl, 0.02 M Tris-HCl, pH 7.4, and centrifuged for 6 h at 46,000 rpm in SW55 rotor at 4 ^o^C. The pellet was dissolved in 200 μL of TNE buffer (10 mM Tris-HCl, pH 7.4; 150 mM NaCl, 1 mM EDTA)-containing 1x Protease Inhibitor Cocktail (CST, #5871) for overnight at 4°C, followed by addition of NP-40 and DTT to the final concentration of 1% and 10 mM, respectively, and incubate at 4°C for 30 min. Dynabeads Protein G beads pre-absorbed with mouse monoclonal antibody against HBV core protein (Santa Cruz, sc-52406) were mixed with the samples and incubated at 4°C for overnight. The beads were washed with IP lysis buffer (Thermo Fisher, 87788) for 8 times. HBV core protein and PP1α/β in the immunocomplex were detected by Western blot assays.

### Phos-tag gel assay

Phos-tag gel containing Phos-tag with zinc ion was purchased from Fujifilm Wako Cure Chemical Corporation (Cat. No. 199–18011). Protein samples were resolved by electrophoresis with Tris-Glycine buffer. The gel was then soaked in transfer buffer containing 10 mM EDTA for 20 min for three times and followed by soaking the gel with transfer buffer without EDTA for 10 min. Proteins in the gel were then transferred to PVDF membrane. The membrane was blocked with 10% milk for 40 min. HBV Cp were detected by probing the membrane with antibody HBc-170A. Bound antibody was revealed by HRP-linked anti-rabbit IgG (CST, #7074S) secondary antibodies and visualized by ChemiDOC Touch Image System (BioRad).

### Endogenous DNA polymerase assay

An endogenous DNA polymerase assay was carried out as described previously [54]. Briefly, intracellular HBV capsids were prepared from AML12HBV10 cell lysates by 30% sucrose cushion ultracentrifugation. The endogenous DNA polymerase reaction was assembled by mixing 20 μl of HBV capsid assembly preparation with 25 μl of 2× endogenous DNA polymerase reaction (EPR) buffer containing 0.15 M NaCl, 0.1 M Tris-HCl (pH 8.0), 20 mM MgCl_2_, 2 mM dithiothreitol, 0.2% (vol/vol) Nonidet P-40. dNTP and protein phosphatase inhibitors were added, as indicated, to 0.1 mM or a specified final concentration, respectively. Water was added to bring the reaction volume to 50 μl. After incubation at 37°C for 16 h, HBV core protein was detected by a western blot assay. Viral DNA were extracted and resolved in 1.5% agarose gel and transferred onto Hybond-XL membrane. The membrane was probed with α-^32^P-UTP labeled minus strand specific full length HBV riboprobe.

## Supporting information

S1 Fig Schematic presentation of HBV Cp domain structures as well as major (red) and minor (blue) phosphorylation sites. The amino acid sequence of Cp CTD is from a genotype D HBV (GenBank Accession No. X01587). The four major cellular kinases that are capable of phosphorylating some or all phosphorylation sites of the CTD are highlighted. The cellular protein phosphatase(s) catalyze Cp dephosphorylation remains to be identified.
(TIF)

S2 Fig PP2CA specific inhibitor LB-100 does not inhibit pgRNA encapsidation and Cp dephosphorylation. (**A**) Experimental schedule: AML12HBVpolY63F cell were cultured in the absence of tet for 4 h and then mock-treated or treated with the indicated concentrations of LB-100 for 12 h. (**B**) Intracellular capsids and encapsidated pgRNA were detected by particle gel assay. Cp phosphorylation status was determined by a Western blot assay. β-actin served as a loading control. (**C** and **D**) The band intensity of capsids, encapsidated RNA as well as hyper- and hypo-phosphorylated Cp in panel B were quantified by Gelpro32 software. The amount of encapsidated pgRNA was normalized to the amount of total capsids in each sample and presented as a fraction of the amount in mock-treated cells (C). The extent of Cp dephosphorylation was expressed as the percentage of hypophosphoryated Cp in total Cp for each sample (D).
(TIF)

S3 Fig Amino acid sequence alignment of human and mouse PP1 catalytic subunit isoforms. The N- and C-terminal variable regions are indicated. The variable residues among the different isoforms are highlighted.
(TIF)

S4 Fig Validation of PP1 siRNA specificity. AML12HBV10 cells were cultured in the presence of tet for 24 h and then tansfected with 10 pmol control siRNA or siRNA targeting the mRNA of three different PP1 catalytic subunit isoforms, PP1α, PP1β and PP1γ, by using Lipofectamine 2000. At 24 h post transfection, cells were cultured in the absence of tet for 48 h and then harvested. (**A**) Intracellular PP1 isoforms were determined by Western blot assays with specific antibodies. β-actin served as a loading control. (**B**) The density of protein bands were quantified by Gelpro32 software. The level of PP1 expression in each sample was normalized β-actin and plotted as a fraction of the amount in cells transfected with control siRNA.
(TIF)

S5 Fig siRNA knockdown of PP2CA expression does not alter HBV Cp dephosphorylation. (A) Experimental schedule: AML12HBVpolY63F cells were cultured in the presence of tet for 24 h and then transfected with 10 pmol control siRNA or siRNA targeting the mRNA of PP2CA by using Lipofectamine 2000. At 24 h post tranfection, cells were cultured in the absence of tet for 48 h and harvested. (**B**) Intracellular PP2CA and HBV Cp was determined by Western blot assays. β-actin served as a loading control.
(TIF)

S6 Fig Encapsidation of PP1α/β was correlated with HBV Cp dephosphorylation in human cells. HepG2 and 293T cells were transfected with the indicated plasmids by using lipofectamine 2000 and harvested at 3 or 2 days post transfection, respectively. The cells were lysed with IP lysis buffer. The cell lysates were clarified by centrifugation at 10,000 g at 4°C for 10 min. The supernatants were subjected for IP with an antibody against HBV core (Santa Cruz) or control IgG. HBV Cp and PP1α/β proteins in the cell lysates (input) and immunocomplexes of IP were detected by Western blot assays with antibody HBc-170A or antibody against PP1α/β.
(TIF)

S7 Fig Selective encapsidation of PP1α/β does not depend on the N- and C-terminal variable region of PP1. HEK 293T cells were co-transfected with the indicated plasmids by using lipofectamine 2000 and harvested at 2 days post transfection (A to C). The plasmids expressing N- and/or C terminal deleted PP1β or chimeric proteins of PP1β and PP1γ are illustrated in the upper panel of (B and C). The cells were lysed with IP lysis buffer. The cell lysates were clarified by centrifugation at 10,000 g at 4°C for 10 min. The supernatants were subjected to IP with an antibody against HBV core (Santa Cruz) or control IgG. HBV Cp and PP1α/β proteins in cell lysates (input) and immunocomplexes of IP were detected by Western blot assays with antibody HBc-170A or antibody against Myc or Flag tag.
(TIF)

S8 Fig PP1α/β was encapsidated into HBV patient viral particle. **(A)** Serum from a HBV carrier or healthy individuals were precipitated through 30% sucrose cushion ultracentrifugation and resolved by SDS-PAGE. HBV Cp and PP1α/β proteins were detected by western blot assay. (**B**) The ultracentrifugation pelleted serum samples were dissolved and treated with 1% NP-40 and 10 mM DTT to remove viral envelope. The viral capsids were precipitated with a mouse monoclonal antibody against HBV core (Santa Cruz). HBV Cp and PP1α/β proteins in immunocomplexes were detected by Western blot assay with antibody HBc-170A or antibody against PP1α/β, respectively. HBV nucelocapsids pelleted by 30% sucrose cushion ultracentrifugation from the lysates of AML12HBVpolY63F cell served as positive controls.
(TIF)

S9 Fig Structure of two HBV core protein allosteric modulators (CpAMs) used in this study. (TIF)

S10 Fig GYH, a type II CpAM, inhibited PP1α/β encapsidation in AML12HBVpolY63F cells. (**A**) Experimental schedule: AML12HBVpolY63F cell was cultured in the absence of tet for 4 h and then left untreated or treated with 100nM GYH-2-18 (GYH) for 20 h. (**B**) Intracellular capsids and encapsidated pgRNA were detected by particle gel assay. (**C**) GYH untreated and treated cells were lysed by IP lysis buffer. AML12 cell lysate was used as a negative control. The cell lysates were subjected for IP with antibody against HBV core or control IgG. HBV Cp and PP1α/β proteins in the cell lysates (input) and immunocomplexes of IP were detected by Western blot assays with antibody HBc-170A or antibody against PP1α and/or β.
(TIF)

S11 Fig Phosphatase inhibitors did not alter Cp phosphorylation status and viral DNA synthesis in an endogenous DNA synthesis assay of purified cytoplasmic capsids. Endogenous DNA polymerase assay was performed in absence or presence of dNTP, with or without PP1 inhibitor tautomycin and PP2CA inhibitor LB-100 as indicated, for 16 h. HBV core protein phosphorylation status were detected by Western blot with antibody HBc-170A (**A**). HBV DNA were extracted and detected by Southern blot hybridization with α-^32^P-UTP labeled full-length plus-strand HBV RNA (**B**).
(TIF)

S1 Table Primers sequences for plasmid construction. (DOCX)

S2 Table DNA fragments synthesized for generation of PP1β with deletion of C-terminal region as well as C-terminal and different N-terminal regions. (DOCX)

S1 Data Excel spreadsheet containing, in separate sheets, the underlying numerical data and statistical analysis for Figure panel 2B, 2D, 3C, 3D, 3E, S2C, S2D and S4B. (XLSX)
