# Supplementary figures and images for: Protein phosphatase 1 catalyzes HBV core protein dephosphorylation and is co-packaged with viral pregenomic RNA into nucleocapsids
Source: PLoS Pathog. 2020 Jul 23;16(7):e1008669. doi: 10.1371/journal.ppat.1008669 (PMC7402523; doi:10.1371/journal.ppat.1008669)

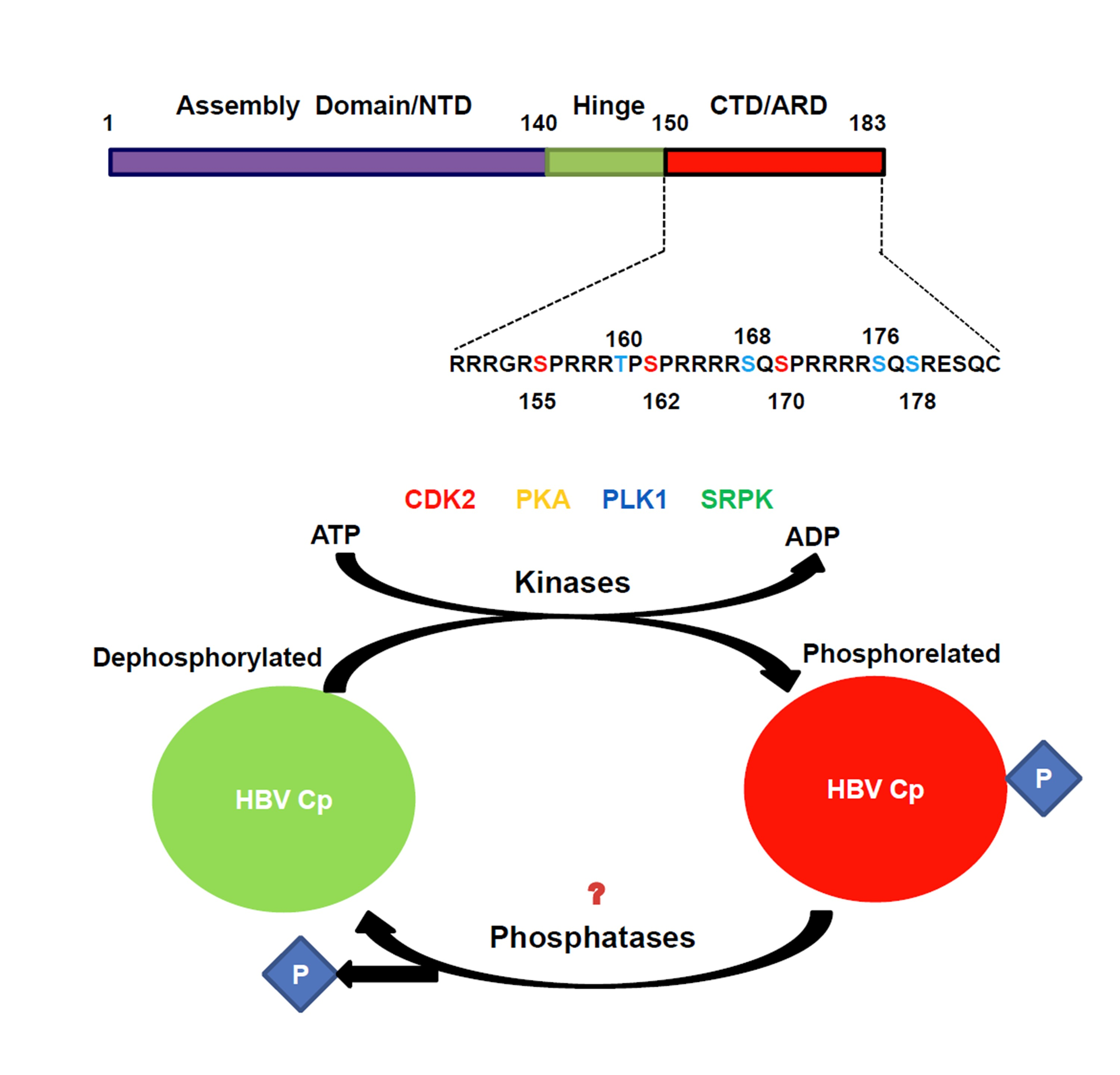

Supplement: S1 Fig — The amino acid sequence of Cp CTD is from a genotype D HBV (GenBank Accession No. X01587). The four major cellular kinases that are capable of phosphorylating some or all phosphorylation sites of the CTD are highlighted. The cellular protein phosphatase(s) catalyze Cp dephosphorylation remains to be identified. (TIF) [file ppat.1008669.s001.tif]

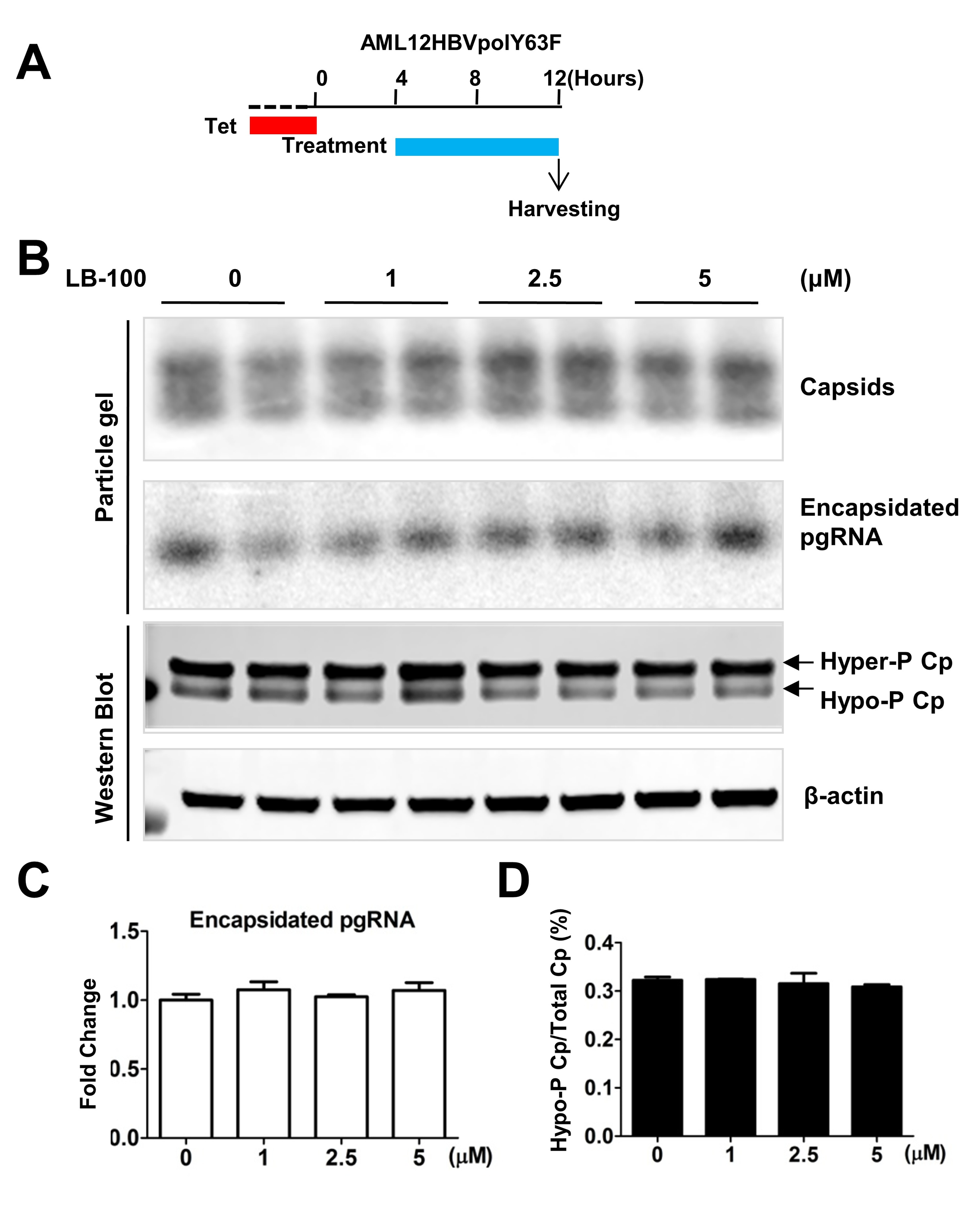

Supplement: S2 Fig — (A) Experimental schedule: AML12HBVpolY63F cell were cultured in the absence of tet for 4 h and then mock-treated or treated with the indicated concentrations of LB-100 for 12 h. (B) Intracellular capsids and encapsidated pgRNA were detected by particle gel assay. Cp phosphorylation status was determined by a Western blot assay. β-actin served as a loading control. (C and D) The band intensity of capsids, encapsidated RNA as well as hyper- and hypo-phosphorylated Cp in panel B were quantified by Gelpro32 software. The amount of encapsidated pgRNA was normalized to the amount of total capsids in each sample and presented as a fraction of the amount in mock-treated cells (C). The extent of Cp dephosphorylation was expressed as the percentage of hypophosphoryated Cp in total Cp for each sample (D). (TIF) [file ppat.1008669.s002.tif]

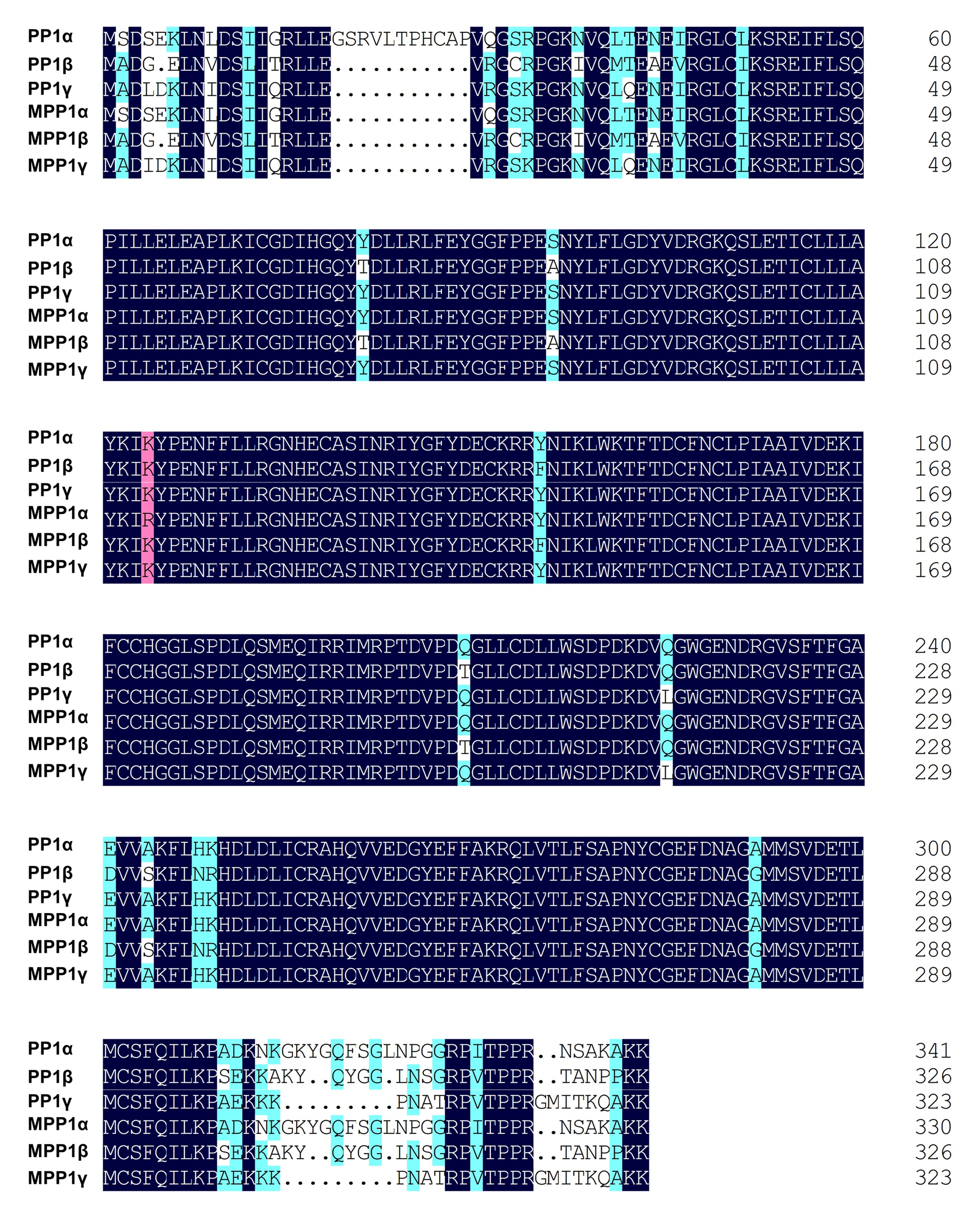

Supplement: S3 Fig — The N- and C-terminal variable regions are indicated. The variable residues among the different isoforms are highlighted. (TIF) [file ppat.1008669.s003.tif]

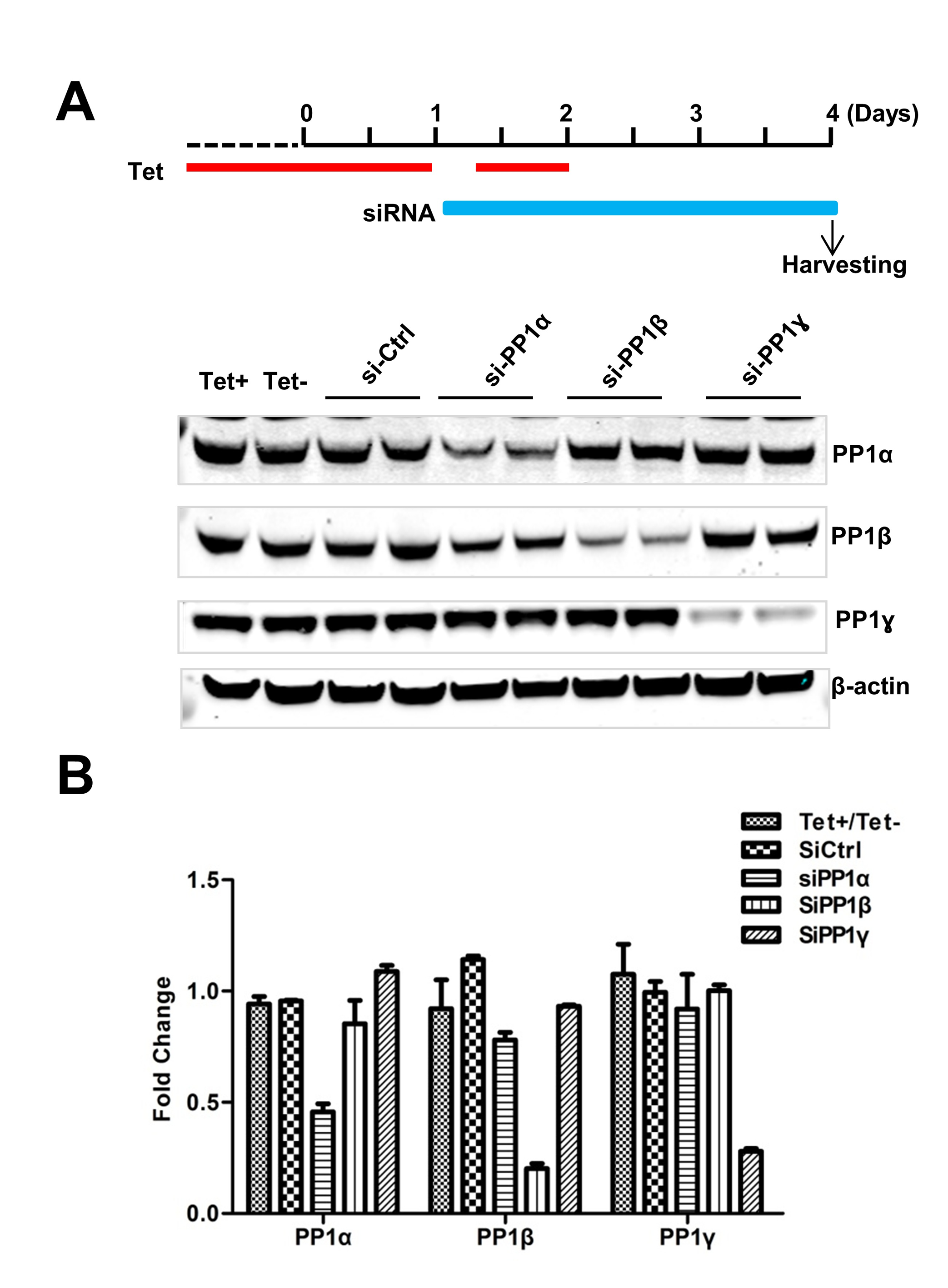

Supplement: S4 Fig — AML12HBV10 cells were cultured in the presence of tet for 24 h and then tansfected with 10 pmol control siRNA or siRNA targeting the mRNA of three different PP1 catalytic subunit isoforms, PP1α, PP1β and PP1γ, by using Lipofectamine 2000. At 24 h post transfection, cells were cultured in the absence of tet for 48 h and then harvested. (A) Intracellular PP1 isoforms were determined by Western blot assays with specific antibodies. β-actin served as a loading control. (B) The density of protein bands were quantified by Gelpro32 software. The level of PP1 expression in each sample was normalized β-actin and plotted as a fraction of the amount in cells transfected with control siRNA. (TIF) [file ppat.1008669.s004.tif]

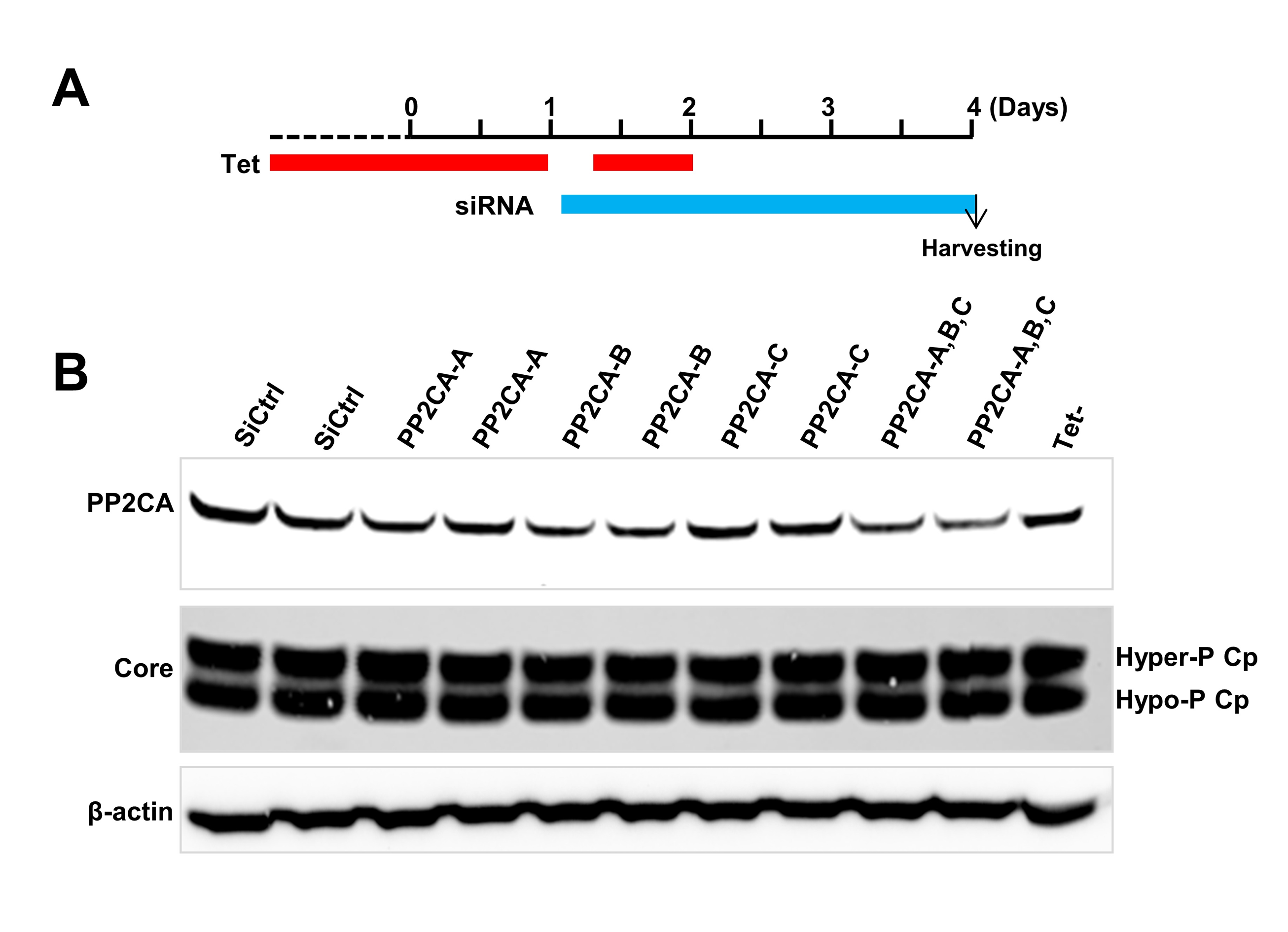

Supplement: S5 Fig — (A) Experimental schedule: AML12HBVpolY63F cells were cultured in the presence of tet for 24 h and then transfected with 10 pmol control siRNA or siRNA targeting the mRNA of PP2CA by using Lipofectamine 2000. At 24 h post tranfection, cells were cultured in the absence of tet for 48 h and harvested. (B) Intracellular PP2CA and HBV Cp was determined by Western blot assays. β-actin served as a loading control. (TIF) [file ppat.1008669.s005.tif]

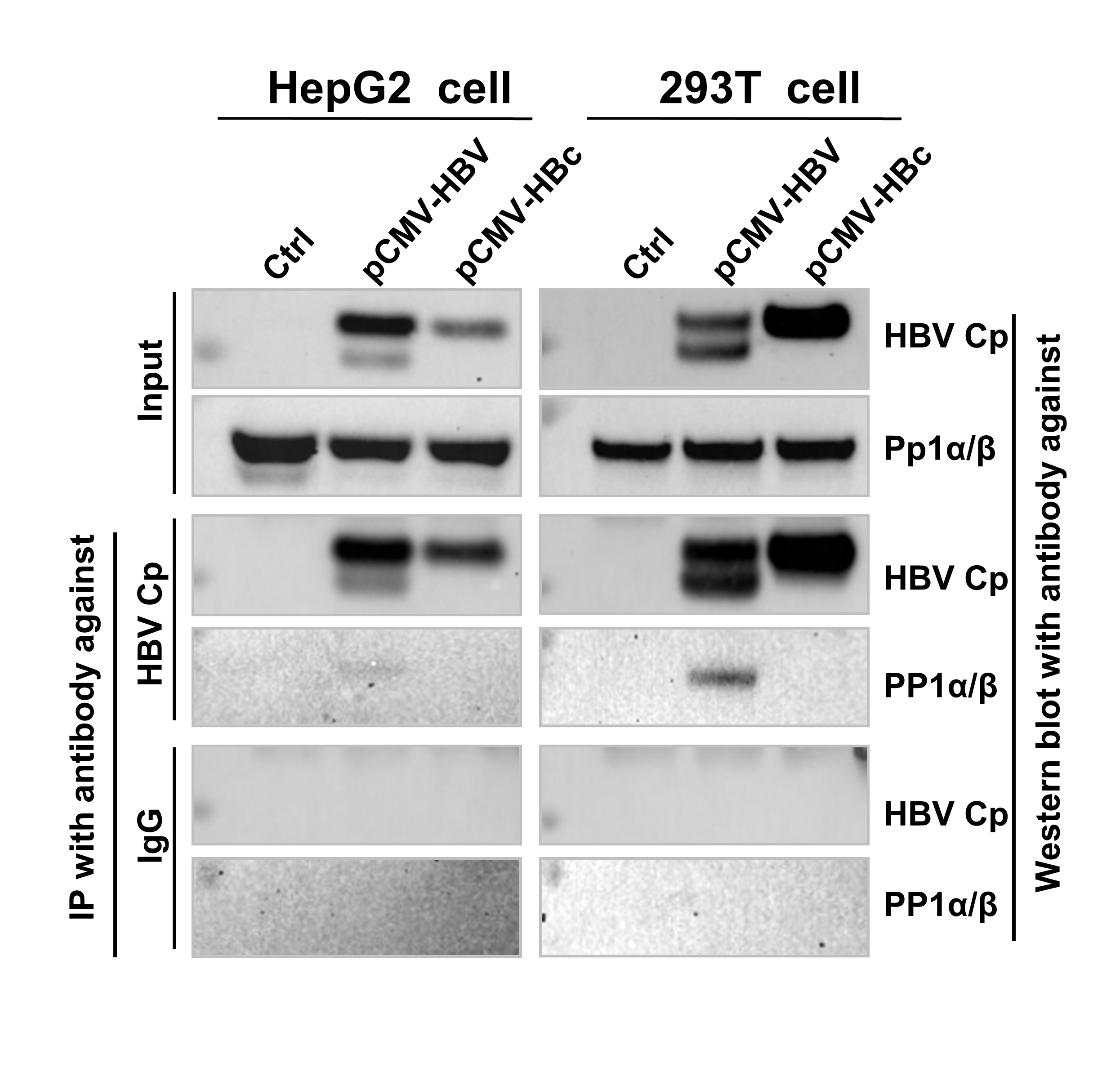

Supplement: S6 Fig — HepG2 and 293T cells were transfected with the indicated plasmids by using lipofectamine 2000 and harvested at 3 or 2 days post transfection, respectively. The cells were lysed with IP lysis buffer. The cell lysates were clarified by centrifugation at 10,000 g at 4°C for 10 min. The supernatants were subjected for IP with an antibody against HBV core (Santa Cruz) or control IgG. HBV Cp and PP1α/β proteins in the cell lysates (input) and immunocomplexes of IP were detected by Western blot assays with antibody HBc-170A or antibody against PP1α/β. (TIF) [file ppat.1008669.s006.tif]

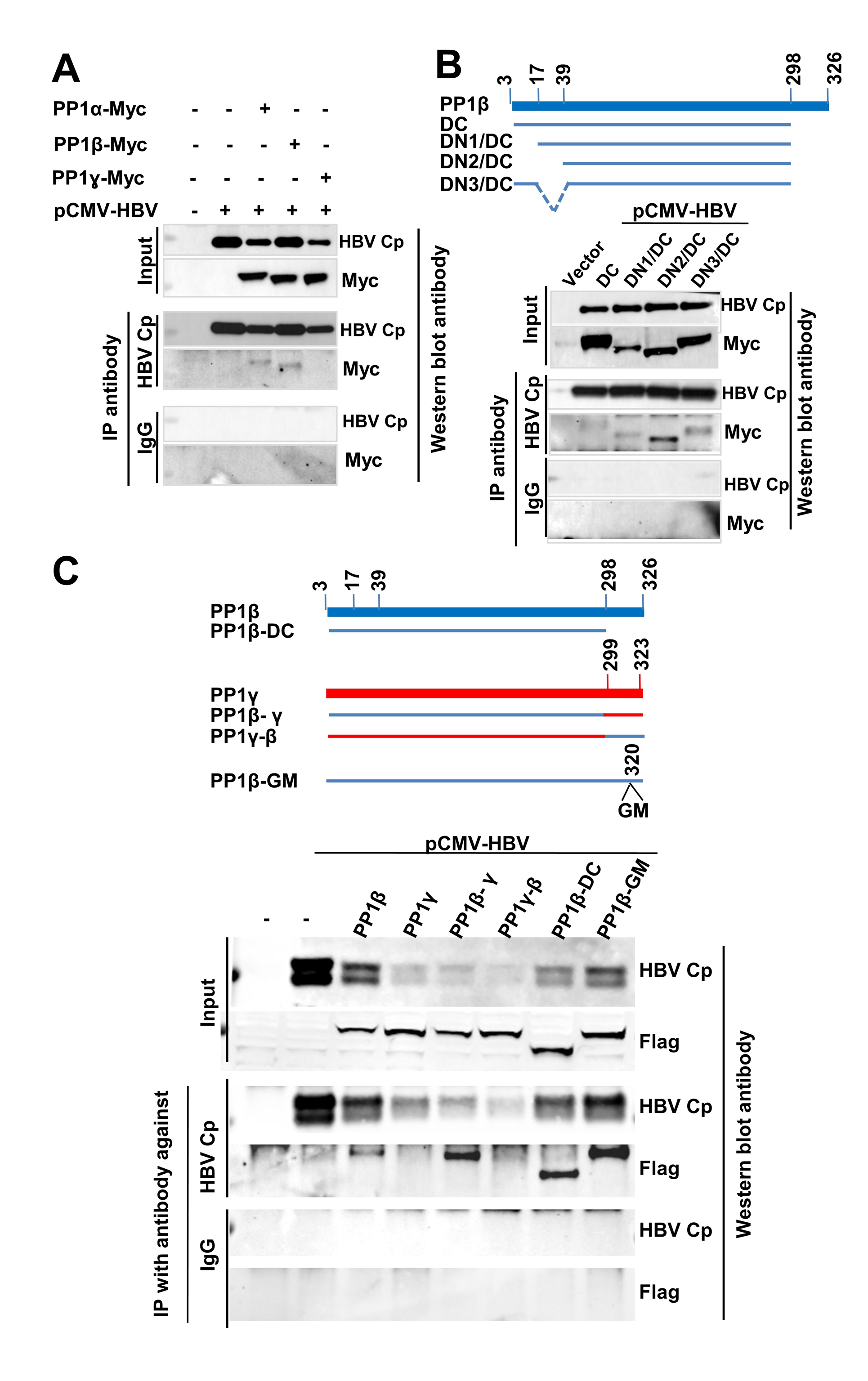

Supplement: S7 Fig — HEK 293T cells were co-transfected with the indicated plasmids by using lipofectamine 2000 and harvested at 2 days post transfection (A to C). The plasmids expressing N- and/or C terminal deleted PP1β or chimeric proteins of PP1β and PP1γ are illustrated in the upper panel of (B and C). The cells were lysed with IP lysis buffer. The cell lysates were clarified by centrifugation at 10,000 g at 4°C for 10 min. The supernatants were subjected to IP with an antibody against HBV core (Santa Cruz) or control IgG. HBV Cp and PP1α/β proteins in cell lysates (input) and immunocomplexes of IP were detected by Western blot assays with antibody HBc-170A or antibody against Myc or Flag tag. (TIF) [file ppat.1008669.s007.tif]

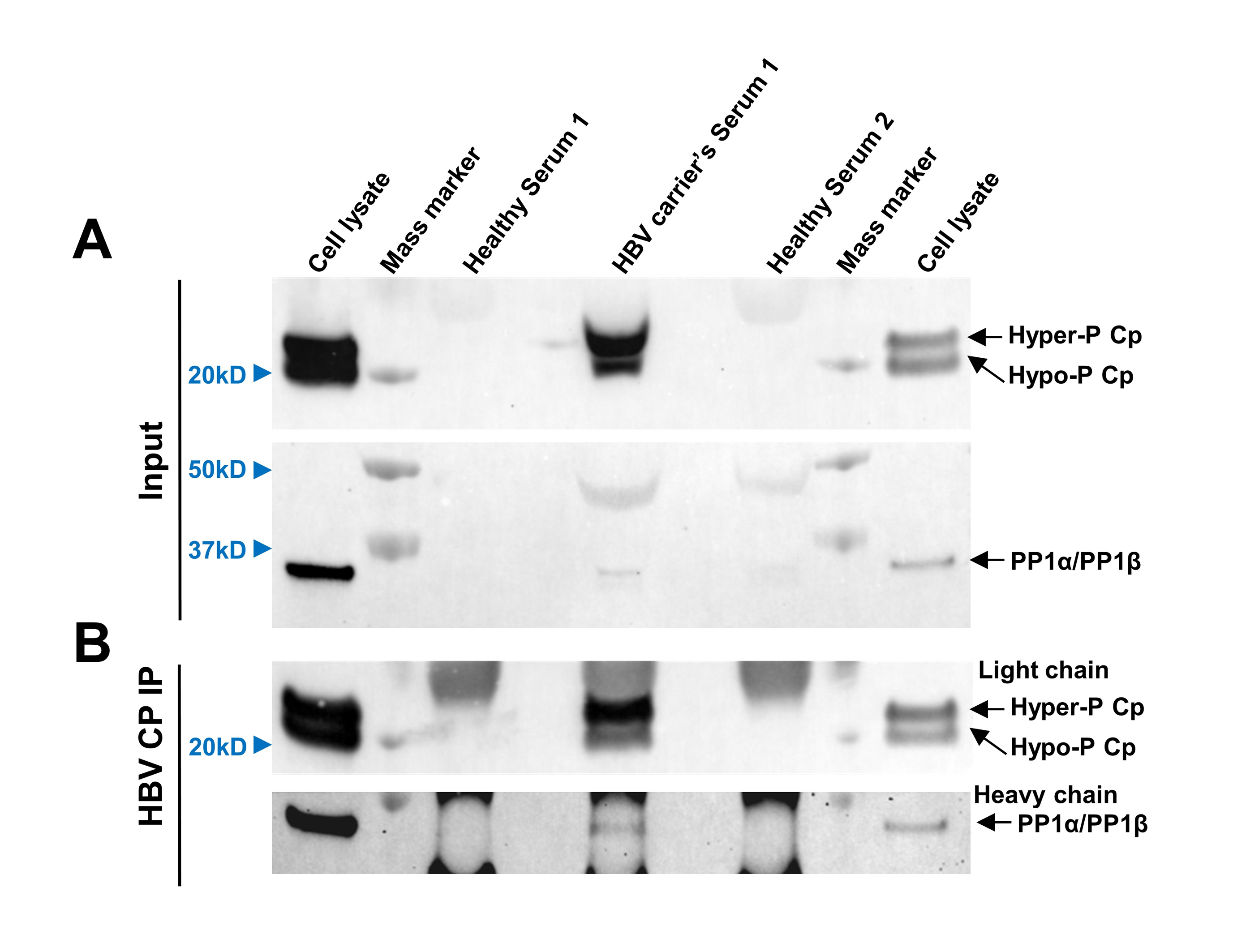

Supplement: S8 Fig — (A) Serum from a HBV carrier or healthy individuals were precipitated through 30% sucrose cushion ultracentrifugation and resolved by SDS-PAGE. HBV Cp and PP1α/β proteins were detected by western blot assay. (B) The ultracentrifugation pelleted serum samples were dissolved and treated with 1% NP-40 and 10 mM DTT to remove viral envelope. The viral capsids were precipitated with a mouse monoclonal antibody against HBV core (Santa Cruz). HBV Cp and PP1α/β proteins in immunocomplexes were detected by Western blot assay with antibody HBc-170A or antibody against PP1α/β, respectively. HBV nucelocapsids pelleted by 30% sucrose cushion ultracentrifugation from the lysates of AML12HBVpolY63F cell served as positive controls. (TIF) [file ppat.1008669.s008.tif]

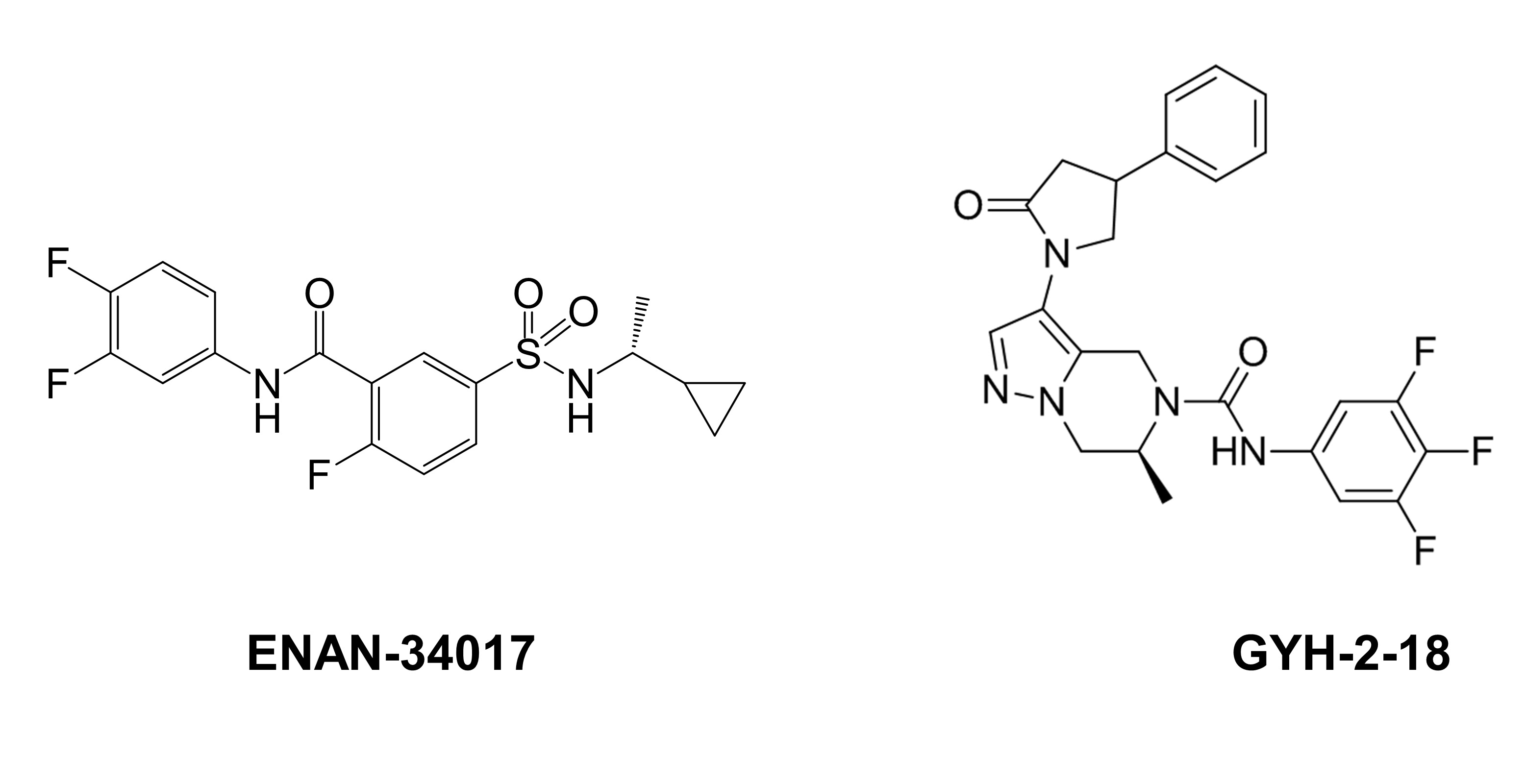

Supplement: S9 Fig — (TIF) [file ppat.1008669.s009.tif]

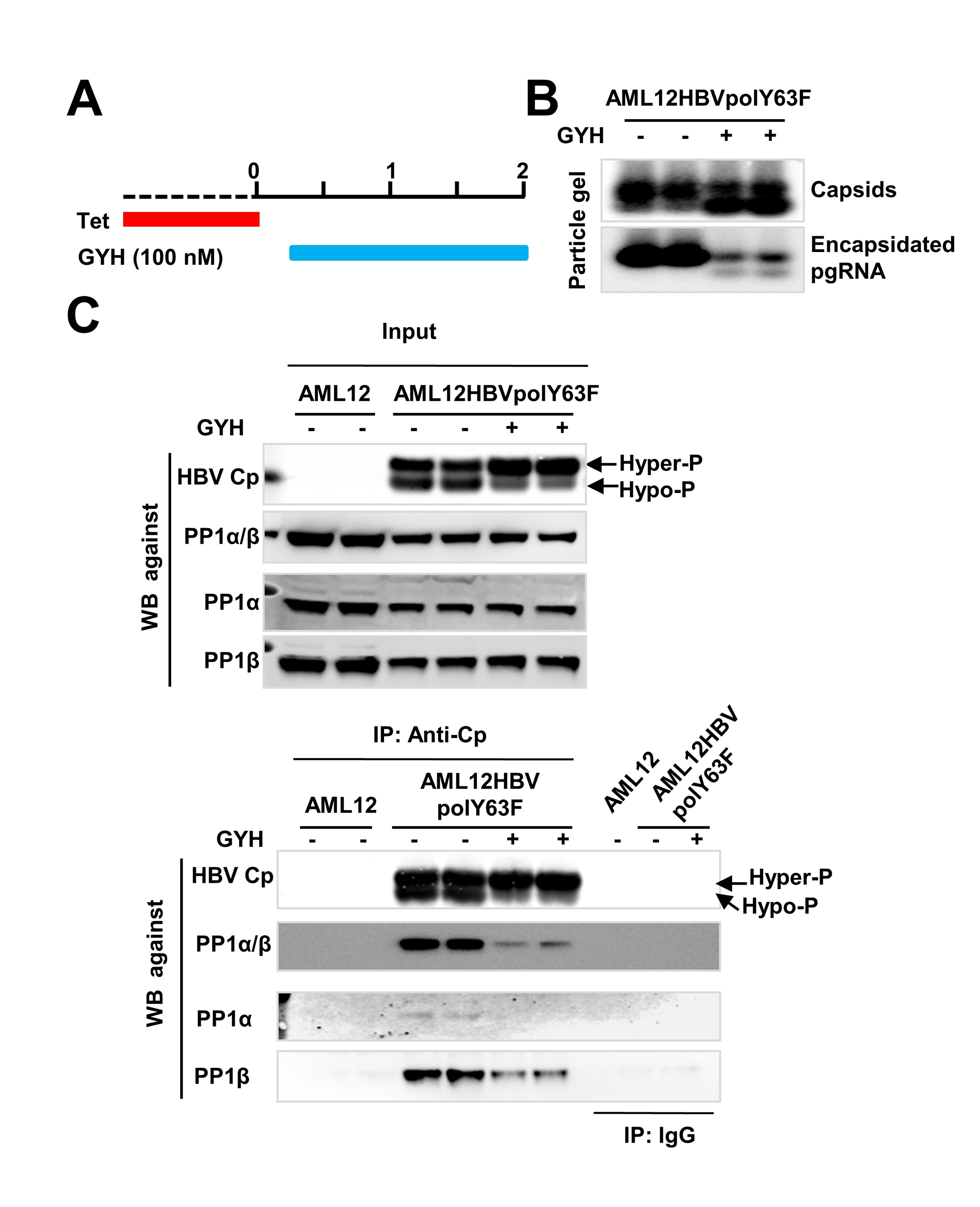

Supplement: S10 Fig — (A) Experimental schedule: AML12HBVpolY63F cell was cultured in the absence of tet for 4 h and then left untreated or treated with 100nM GYH-2-18 (GYH) for 20 h. (B) Intracellular capsids and encapsidated pgRNA were detected by particle gel assay. (C) GYH untreated and treated cells were lysed by IP lysis buffer. AML12 cell lysate was used as a negative control. The cell lysates were subjected for IP with antibody against HBV core or control IgG. HBV Cp and PP1α/β proteins in the cell lysates (input) and immunocomplexes of IP were detected by Western blot assays with antibody HBc-170A or antibody against PP1α and/or β. (TIF) [file ppat.1008669.s010.tif]

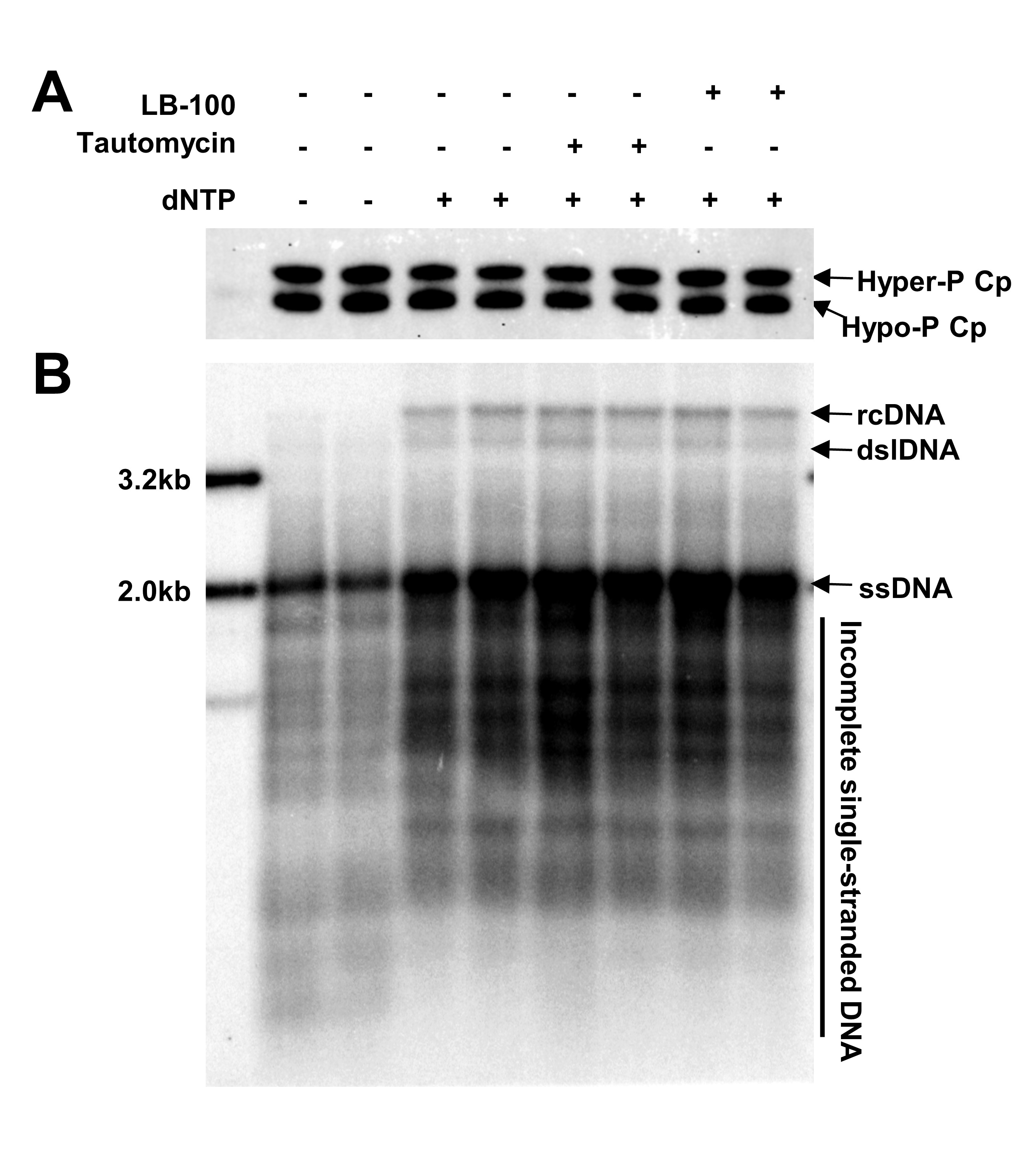

Supplement: S11 Fig — Endogenous DNA polymerase assay was performed in absence or presence of dNTP, with or without PP1 inhibitor tautomycin and PP2CA inhibitor LB-100 as indicated, for 16 h. HBV core protein phosphorylation status were detected by Western blot with antibody HBc-170A (A). HBV DNA were extracted and detected by Southern blot hybridization with α-32P-UTP labeled full-length plus-strand HBV RNA (B). (TIF) [file ppat.1008669.s011.tif]
