# Supplementary material for: Protein phosphatase 1 catalyzes HBV core protein dephosphorylation and is co-packaged with viral pregenomic RNA into nucleocapsids
Source: PLoS Pathog. 2020 Jul 23;16(7):e1008669. doi: 10.1371/journal.ppat.1008669 (PMC7402523; doi:10.1371/journal.ppat.1008669)
Supplement: S1 Table — (DOCX) [file ppat.1008669.s012.docx]

**S1 Table. Primers sequences**­­­­­­­­­ **for plasmid construction.** _______________________________________________________

­­­­­­­­­­­­­­­­­Primer Sequence (5’ – 3’)

­­­­­­­­­­_______________________________________________________

F1 AAGTCGAGCTCGGTACCCGGGTCGA

R1 AAAGGTTGTGGAATTCCACTGCATG

F105 TTACTGGTCTTTTTTCTTCTACTGTACC

R105 GGTACAGTAGAAGAAAAAAGACCAGTAA

CoreF TACCGC **GGATCC** ATGGACATCGACCCTTATA

CoreR TAGCCC **AAGCTT** CTAACATTGAGGTTCCCG

_______________________________________________________
