# Supplementary material for: Protein phosphatase 1 catalyzes HBV core protein dephosphorylation and is co-packaged with viral pregenomic RNA into nucleocapsids
Source: PLoS Pathog. 2020 Jul 23;16(7):e1008669. doi: 10.1371/journal.ppat.1008669 (PMC7402523; doi:10.1371/journal.ppat.1008669)
Supplement: S2 Table — (DOCX) [file ppat.1008669.s013.docx]

**S2 Table. DNA fragments synthesized for generation of PP1β with deletion of C-terminal region as well as C-terminal and different N-terminal regions**

| DNA  fragment | Synthetic DNA |  |
| --- | --- | --- |
| DC | GGATATGAATTTTTTGCTAAACGACAGTTGGTAACCTTATTTTCAGCCCCAAATTACTGTGGCGAGTTTGATAATGCTGGTGGAATGATGAGTGTGGATGAAACTTTGATGTGTTCATTTCAGATATTGAAACCAACGCGGCCGCTCGAGCAGAAACTCATCTCAGAAGAGGATCTGGCA |  |
| DN1 | CGTCGACTGGATCCGGTACCGAGGAGATCTGCCGCCGCGATCGCCATGGCGCGGCTGCTGGAGGTACGAGGATGTCGTCCAGGAAAGATTGTGCAGATGACTGAAGCAGAAGTTCGAGGCTTATGTATCAAGTCTCGGGAGATCTTTCTCAGCCAGCCTATTCTTTTGGAATTGGAAGCACCGCTGAAAATTTGTGGAGATATTCATGGACAGTATACAGATTTACTGAGATTATTTGAATATGGAGGTTTCCCACCAGAAGCCAACTATCTTTTCTTAGGAGATTATGTGGACAGAGGAAAGCAGTCTTTGGAAACCATTTGTTTGCTATTGGCTTATAAAATCAAATATCCAGAGAACTTCTTTCTCTTAAGAGGAAACCATGAGTGTGCTAGCATCAATCGCATTTATGGATTCT |  |
| DN2 | CGTCGACTGGATCCGGTACCGAGGAGATCTGCCGCCGCGATCGCCATGGCGGTGCAGATGACTGAAGCAGAAGTTCGAGGCTTATGTATCAAGTCTCGGGAGATCTTTCTCAGCCAGCCTATTCTTTTGGAATTGGAAGCACCGCTGAAAATTTGTGGAGATATTCATGGACAGTATACAGATTTACTGAGATTATTTGAATATGGAGGTTTCCCACCAGAAGCCAACTATCTTTTCTTAGGAGATTATGTGGACAGAGGAAAGCAGTCTTTGGAAACCATTTGTTTGCTATTGGCTTATAAAATCAAATATCCAGAGAACTTCTTTCTCTTAAGAGGAAACCATGAGTGTGCTAGCATCAATCGCATTTATGGATTCT |  |
| DN3 | CGTCGACTGGATCCGGTACCGAGGAGATCTGCCGCCGCGATCGCCATGGCGGACGGGGAGCTGAACGTGGACAGCCTCATCACCCGGCTGCTGGAGGTACGAGGATGTCGTCCAGGAAAGATTGTGCAGAAGTCTCGGGAGATCTTTCTCAGCCAGCCTATTCTTTTGGAATTGGAAGCACCGCTGAAAATTTGTGGAGATATTCATGGACAGTATACAGATTTACTGAGATTATTTGAATATGGAGGTTTCCCACCAGAAGCCAACTATCTTTTCTTAGGAGATTATGTGGACAGAGGAAAGCAGTCTTTGGAAACCATTTGTTTGCTATTGGCTTATAAAATCAAATATCCAGAGAACTTCTTTCTCTTAAGAGGAAACCATGAGTGTGCTAGCATCAATCGCATTTATGGATTCTATGATGAATGCAAACGAAGATTTAATATTAAATTG | |
| PP1β-γ | GGATATGAATTTTTTGCTAAACGACAGTTGGTAACCTTATTTTCAGCCCCAAATTACTGTGGCGAGTTTGATAATGCTGGTGGAATGATGAGTGTGGATGAAACTTTGATGTGTTCATTTCAGATATTGAAACCAGCAGAGAAAAAGAAGCCAAATGCCACGAGACCTGTAACGCCTCCAAGGGGTATGATCACAAAGCAAGCAAAGAAAACGCGGCCGCTCGAGCAGAAACTCATCTCAGAAGAGGATCTGGCA | |
| PP1γ-β | GAGTTTGACAATGCAGGTGCCATGATGAGTGTGGATGAAACACTAATGTGTTCTTTTCAGATTTTAAAGCCTTCTGAAAAGAAAGCTAAATACCAGTATGGTGGACTGAATTCTGGACGTCCTGTCACTCCACCTCGAACAGCTAATCCGCCGAAGAAAAGGACGCGGCCGCTCGAGCAGAAACTCATCTCAGAAGAGGATCTGGCA | |
| PP1β-GM | GGATATGAATTTTTTGCTAAACGACAGTTGGTAACCTTATTTTCAGCCCCAAATTACTGTGGCGAGTTTGATAATGCTGGTGGAATGATGAGTGTGGATGAAACTTTGATGTGTTCATTTCAGATATTGAAACCATCTGAAAAGAAAGCTAAATACCAGTATGGTGGACTGAATTCTGGACGTCCTGTCACTCCACCTCGAGGTATGACAGCTAATCCGCCGAAGAAAAGGACGCGGCCGCTCGAGCAGAAACTCATCTCAGAAGAGGATCTGGCA | |
